# Hydrogen sulfide inhibits calcification of heart valves; implications for calcific aortic valve disease

**DOI:** 10.1111/bph.14691

**Published:** 2019-04-24

**Authors:** Katalin Éva Sikura, László Potor, Tamás Szerafin, Melinda Oros, Péter Nagy, Gábor Méhes, Zoltán Hendrik, Abolfazl Zarjou, Anupam Agarwal, Niké Posta, Roberta Torregrossa, Matthew Whiteman, Ibolya Fürtös, György Balla, József Balla

**Affiliations:** ^1^ HAS‐UD Vascular Biology and Myocardial Pathophysiology Research Group Hungarian Academy of Sciences Debrecen Hungary; ^2^ Department of Medicine, Faculty of Medicine University of Debrecen Debrecen Hungary; ^3^ Department of Pediatrics, Faculty of Medicine University of Debrecen Debrecen Hungary; ^4^ Department of Medicine, Division of Nephrology, Nephrology Research and Training Center and Center for Free Radical Biology University of Alabama at Birmingham Birmingham Alabama; ^5^ Department of Pathology University of Debrecen, Faculty of Medicine Debrecen Hungary; ^6^ Department of Cardiac Surgery, Faculty of Medicine University of Debrecen Debrecen Hungary; ^7^ Department of Molecular Immunology and Toxicology National Institute of Oncology Budapest Hungary; ^8^ College of Medicine and Health University of Exeter Medical School Exeter UK

## Abstract

**Background and Purpose:**

Calcification of heart valves is a frequent pathological finding in chronic kidney disease and in elderly patients. Hydrogen sulfide (H_2_S) may exert anti‐calcific actions. Here we investigated H_2_S as an inhibitor of valvular calcification and to identify its targets in the pathogenesis.

**Experimental Approach:**

Effects of H_2_S on osteoblastic transdifferentiation of valvular interstitial cells (VIC) isolated from samples of human aortic valves were studied using immunohistochemistry and western blots. We also assessed H2S on valvular calcification in apolipoprotein E‐deficient (ApoE^−/−^) mice.

**Key Results:**

In human VIC, H_2_S from donor compounds (NaSH, Na_2_S, GYY4137, AP67, and AP72) inhibited mineralization/osteoblastic transdifferentiation, dose‐dependently in response to phosphate. Accumulation of calcium in the extracellular matrix and expression of osteocalcin and alkaline phosphatase was also inhibited. RUNX2 was not translocated to the nucleus and phosphate uptake was decreased. Pyrophosphate generation was increased via up‐regulating ENPP2 and ANK1. Lowering endogenous production of H_2_S by concomitant silencing of cystathionine γ‐lyase (CSE) and cystathionine β‐synthase (CBS) favoured VIC calcification. analysis of human specimens revealed higher Expression of CSE in aorta stenosis valves with calcification (AS) was higher than in valves of aortic insufficiency (AI). In contrast, tissue H_2_S generation was lower in AS valves compared to AI valves. Valvular calcification in ApoE^−/−^ mice on a high‐fat diet was inhibited by H_2_S.

**Conclusions and Implications:**

The endogenous CSE‐CBS/H_2_S system exerts anti‐calcification effects in heart valves providing a novel therapeutic approach to prevent hardening of valves.

**Linked Articles:**

This article is part of a themed section on Hydrogen Sulfide in Biology & Medicine. To view the other articles in this section visit http://onlinelibrary.wiley.com/doi/10.1111/bph.v177.4/issuetoc

## Graphical abstract

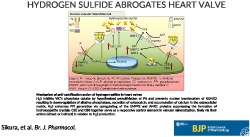

**Abbreviations**
3‐MST: 3‐mercaptopyruvate sulfurtransferase
AI: isolated aortic valve with insufficiency
ALP: alkaline phosphatase
AOAA: aminooxyacetic acid
AP67: (4‐methoxyphenyl)(pyrrolidin‐1‐yl)phosphinodithioc acid)
AP72: 4‐methoxyphenyl piperidinylphosphinodithioc acid
ApoE^−/−^ mice: apolipoprotein E‐deficient mice
AS: stenotic aortic valve with calcification
CAVD: calcific aortic valve disease
CBS: cystathionine β‐synthase
CSE: cystathionine γ‐lyase
ENPP2: ectonucleotide pyrophosphatase/PDE family member 2
GYY4137: (*P*‐(4‐methoxyphenyl)‐*P*‐4‐morpholinylphosphinodithioic acid morpholine salt)
KGA: α‐ketoglutaric acid
PPG: DL propargylglycine
PPi: pyrophosphate
RUNX2: runt‐related transcription factor 2
STED: stimulated emission depletion nanoscopy
VIC: valvular interstitial cells

1 **What is already known**

- Hydrogen sulfide (H_2_S) inhibits osteoblastic transformation of vascular smooth muscle cells.

2 **What this study adds**

- CSE‐ and CBS‐derived H_2_S and H_2_S‐releasing donors inhibit mineralization of aortic valves.
- Anti‐calcification occurs via inhibiting phosphate uptake, preventing nuclear translocation of RUNX2, and increasing pyrophosphate levels.

3 **What is the clinical significance**

- H_2_S‐releasing donors and CSE/CBS‐derived H_2_S have therapeutic potential in calcific aortic valve disease.

## 1 INTRODUCTION

In the developed countries, calcific aortic valve disease (CAVD), an actively regulated disease process, is the most common valvular heart disease with high morbidity and mortality (Yutzey et al., 2014). Numerous studies show that CAVD is accompanied by calcification, lipid accumulation, and inflammation resulting in heterogeneous lesions within the heart valvular tissue. The ratio of the calcified and non‐calcified regions could guide in distinguishing the state of calcification (Chester, 2011; Lusis, Mar, & Pajukanta, 2004; Mohler, 2004; Mohler et al., 2001; Speer & Giachelli, 2004).

Cardiovascular calcification is a complex, chronic disease of the major and medium‐sized arteries including those in aortic valves. Such calcification is involved in the increased risk of cardiovascular morbidity and mortality. Pathologically, it is well known that its progression is much more pronounced in patients with diabetes and chronic kidney disease (CKD; Davignon & Ganz, 2004; Libby, Ridker, & Maseri, 2002; Rajamannan et al., 2011; Stocker & Keaney, 2004).

http://www.guidetopharmacology.org/GRAC/LigandDisplayForward?ligandId=9532 (H_2_S) is the third endogenous gasotransmitter, along with http://www.guidetopharmacology.org/GRAC/LigandDisplayForward?ligandId=2509 and http://www.guidetopharmacology.org/GRAC/LigandDisplayForward?ligandId=9531 (Wang, 2002). Previously, our laboratory demonstrated that http://www.guidetopharmacology.org/GRAC/LigandDisplayForward?ligandId=6278 (a H_2_S donor compound) significantly inhibits the mineralization of vascular smooth muscle cells (Zavaczki et al., 2011). Jiang, Wu, Li, Geng, & Tang (2005) and now many groups have shown http://www.guidetopharmacology.org/GRAC/FamilyDisplayForward?familyId=279#1446 in cardiac tissue and it is important for normal heart function (Chen, Xin, & Zhu, 2007). They demonstrated that disturbance of H_2_S production contributed to the development of heart diseases as manifested by lower levels of H_2_S in plasma, in patients with coronary heart disease (Jiang et al 2005; Shen, Shen, Luo, Guo, & Zhu, 2015). Furthermore, Abe and Kimura (1996) presented for the first time that endogenous H_2_S production by http://www.guidetopharmacology.org/GRAC/FamilyDisplayForward?familyId=279#1443 contributed to normal brain function.

Kang, Neill, and Xian (2017) synthesized a new generation, slow release, H_2_S donor compound (http://www.guidetopharmacology.org/GRAC/LigandDisplayForward?ligandId=9808) from Lawesson's reagent and morpholine. Protonation of GYY4137 resulted in more stable H_2_S‐releasing compounds such as AP67 and AP72. AP72 has an excellent water solubility and a very slow generation of H_2_S, compared to the fast H_2_S‐releasing donors such as NaSH and Na_2_S (Chitnis et al., 2013; Kang et al., 2017; Nagy et al., 2014).

In calcified valves, the valvular interstitial cells (VIC) transdifferentiate into osteoblast‐like cells, identified by up‐regulation of alkaline phosphatase (ALP) activity, and increased levels of osteocalcin expression at later stages (Rajamannan et al., 2003). One of the most potent, recognized inducers of vascular calcification is raised levels of plasma phosphate, in CKD patients (Becs et al., 2016). The increase in its intracellular level promotes nuclear translocation of the osteogenic transcription factor RUNX2 resulting in transition of cells towards an osteoblast phenotype (Ducy, Zhang, Geoffroy, Ridall, & Karsenty, 1997). Phosphate uptake occurs via phosphate carriers http://www.guidetopharmacology.org/GRAC/FamilyDisplayForward?familyId=195#1017 and http://www.guidetopharmacology.org/GRAC/FamilyDisplayForward?familyId=195#1018 (Crouthamel et al., 2013; X. Li, Yang, & Giachelli, 2006).

Pyrophosphate (PPi) is a key regulator of tissue calcification via inhibition of mineralization by binding to nascent hydroxyapatite crystals. Ectonucleotide pyrophosphatase/PDE‐2 (http://www.guidetopharmacology.org/GRAC/ObjectDisplayForward?objectId=2901) is a cell membrane glycoprotein that generates PPi via cleaving http://www.guidetopharmacology.org/GRAC/LigandDisplayForward?ligandId=1713. Ankyrin G1 (ANK1) is a transmembrane protein which has a critical role in the regulation of pyrophosphate levels. The main function of ANK1 is transporting intracellular PPi into the extracellular space (Jansen et al., 2012; Mitton‐Fitzgerald, Gohr, Bettendorf, & Rosenthal, 2016).

The purpose of this study was to explore the effects of H_2_S donors (NaSH, Na_2_S, GYY4137, AP67, or AP72) on the development of calcification in human VIC and ApoE^−/−^ mice fed with atherogenic diet and to investigate its underlying mechanism with regard to the development of atherosclerosis and mineralization. Our study identifies a novel potential treatment for preventing and/or reversing valvular calcification.

## 2 METHODS

### 2.1 Human tissue samples and cell isolation

Human aortic valve leaflets were obtained from patients undergoing valve replacement for stenosis with calcification (AS; *N* = 52) and patients who had severe insufficiency without calcification (AI; *N* = 28). The specimens were collected from January 2015 to December 2017 (80 patients; Regional Research Ethical Committee, Project No.: 61538‐2/2017/EKU and 4699‐2016). VIC were isolated from human heart valves using collagenase (600 U·ml^−1^; Worthington Biochemical Corp.). Cells isolated from donors were employed at passages 2 to 4. All experiments were performed on cells derived from five different donors.

### 2.2 Animals

All animal care and experimental procedures complied with the guidelines from Directive 2010/63/EU of the European Parliament on the protection of animals used for scientific purposes, and were approved by the Scientific and Research Ethics Committee of the Scientific Council of Health of the Hungarian Government under the registration number of DE MÁB/157‐5/2010. Animal studies are reported in compliance with the ARRIVE guidelines (Kilkenny et al., 2010) and with the recommendations made by the British Journal of Pharmacology.

C57BL/6 ApoE^−/−^ mice (The Jackson Laboratory; B6.129P2 Apoetm^1Unc/J^; RRID:IMSR_JAX:002052) were maintained at the University of Debrecen under specific pathogen‐free conditions in accordance with the guidelines from the Institutional Ethical Committee. Mice were randomly divided into three groups. Non‐high fat diet group (*N* = 5) received a standard chow diet. To induce aortic valve calcification, mice were given an atherogenic diet (15% fat, 1.25% cholesterol, ssniff Spezialdiäten GmbH, Soest, Germany) from the age of 8 weeks. The detailed composition of the atherogenic food (high‐fat diet) was as follows: crude nutrients (%): crude protein 19%, crude fat 15.2%, crude fibre 3.4%, crude ash 6.3%, starch 25.6%, sugar 11.2%; additives (per kg): vitamin A 15,000 IU, vitamin D3 1,000 IU, vitamin E 110 mg, vitamin K3 5 mg, vitamin C 0 mg, copper 13 mg. While on the atherogenic diet, mice were injected intraperitoneally with AP72 (266‐μmol·kg^−1^; *N* = 5) or vehicle (saline; *N* = 9) on every other day as previously described (Potor et al., 2018). Aortas were collected after 8 weeks of treatment. All mice were killed by a predictable and controllable administering slow‐fill compressed CO_2_ asphyxiation.

### 2.3 Induction of calcification, calcium measurement, and Alizarin Red S staining

The VIC were cultured in a calcification medium (2.5‐mmol·L^−1^ inorganic phosphate and 1.8‐mmol·L^−1^ calcium–chloride) with or without phenol red for 5 days. Calcium content of the supernatants was determined by QuantiChrome Calcium Assay Kit (Gentaur), normalized to protein content, and expressed as μg·mg^−1^ protein. Alizarin Red S staining was used to visualize the calcium deposition. Plates were fixed with 3.7% formaldehyde for 10 min followed by staining with a 2% solution of Alizarin Red S. Pictures from the stained cells were taken with a light microscope (×10 magnification; Leica DMIL LED microscope).

### 2.4 Quantification of osteocalcin

elisa kit was used (Bender MedSystem) for the quantification of the osteocalcin from EDTA‐solubilized extracellular matrix.

### 2.5 ALP staining

To visualize ALP activity, cells were cultured in 24‐well plates and fixed in citrate–acetone solution (2:3) followed by staining with Naphtanol AS‐MX Fast Violet B solution (Sigma). Light microscope pictures were taken from the different treatments (Leica DMIL LED microscope, Leica DMC4500 camera with Leica application suite LAS Software 4.9.0).

### 2.6 Cell viability assay

Cells were cultured on 0.2% collagen type I coated coverslip. NUCLEAR‐ID® Blue/Red cell viability reagent was added to the cells at dilution 1:1,000 for 30 min at 37°C. Then, the cells were fixed with Fluorescent Mounting Medium (Dako) on Superfrost Ultra Plus Microscope Slide (Thermo Scientific). Images were obtained with an immunofluorescence microscope (Leica DM2500 microscope, Leica DFC480 camera).

### 2.7 Immunofluorescence staining

The antibody‐based procedures used in this study comply with the recommendations made by the *British Journal of Pharmacology*. Cells were cultured on the coverslip and treated with or without calcification medium, with or without phenol red, supplemented with 20‐μmol·L^−1^ AP72 for 24 hr and for 2 days. After treatment, the cells were fixed with 3.7% formaldehyde for 15 min. After fixation, cells were blocked with 10% goat serum for 1 hr at room temperature. Rabbit polyclonal anti‐human RUNX2 (Proteintech Group Cat# 20700‐1‐AP, RRID:AB_2722783) was used (dilution 1:600) as a primary antibody to show RUNX2 localization in VIC. A primary antibody labelled with goat anti‐rabbit Alexa 488 (Molecular Probes Cat# A‐11070, RRID:AB_142134) fluorophore at dilution 1:500 for 1 hr in dark at room temperature. Hoechst was used to stain nuclei. Multicolor STED imaging was acquired with STED (stimulated emission depletion) Leica TCS SP8 gated STED‐CW nanoscopy (Leica Microsystem Mannheim, Germany). Gated STED images were deconvolved using Huygens Professional (Scientific Volume Imaging B.V., Hilversum, Netherlands) software.

### 2.8 Nuclear and cytoplasmic protein extraction

Cells were cultured in growth medium and treated with or without calcification medium supplemented with 20‐μmol·L^−1^ AP72. After treatment, cells were harvested with cell scraper and collected into a centrifuge tube. Pellets were washed twice with PBS and then ice‐cold 1× cytoplasmic lysis buffer (20‐mmol·L^−1^ Tris–HCl pH 8.0, 100‐mmol·L^−1^ NaCl, 300‐mmol·L^−1^ sucrose, 3‐mmol·L^−1^ MgCl_2_, protease inhibitor cocktail) was added to the pellets. Cell suspensions were incubated on ice for 15 min. After centrifugation, the supernatants were collected (contains cytoplasmic proteins); the pellets were washed with PBS and resuspended in ice‐cold nuclear extraction buffer (20‐mmol·L^−1^ Tris–HCl pH 8.0, 300‐mmol·L^−1^ NaCl, 2‐mmol·L^−1^ EDTA pH 8.0, protease inhibitor cocktail). After that, the samples were passed five times through a 27 gauge needle, for the extraction of the nuclear proteins followed by centrifugation at 8,000× *g*, 4°C for 20 min. The supernatant contains the nuclear fraction. The protein concentration of the samples was determined by the BCA Protein Detection Kit (Amersham).

### 2.9 Western blot

To show RUNX2 translocation into the nucleus, we separated the nuclei and cytoplasm fractions with the nuclear extraction kit. After that, nucleus and cytoplasm lysates were separated by electrophoresis with 10% SDS‐PAGE. After blotting, the membrane was incubated with rabbit anti‐human RUNX2 (Cbfa‐1) antibody at 1:600 dilution (Proteintech Group Cat# 20700‐1‐AP, RRID:AB_2722783) for overnight, followed by HRP‐labelled anti‐rabbit IgG antibody incubation for 1 hr at room temperature. The membranes were reprobed with Lamin B1 (Proteintech Group Cat# 12987‐1‐AP, RRID:AB_2136290) and also GAPDH (Novus Cat# NB300‐221, RRID:AB_10077627). Briefly, to evaluate Pit1, Pit2, CSE, and CBS protein expressions from valve tissues, and from the cell, the lysates were electrophoresed with 10% SDS‐PAGE. Western blotting was performed with the following primary antibodies: rabbit anti‐human Pit1 at dilution 1:500 (Abcam Cat# ab24756, RRID:AB_448292), rabbit anti‐human Pit2 at 1:500 dilution (Proteintech Group Cat# 12820‐1‐AP, RRID:AB_2191004), rabbit anti‐human CSE at dilution 1: 600 (Proteintech Group Cat# 12217‐1‐AP, RRID:AB_2087497), rabbit anti‐human CBS at dilution 1:600 (Proteintech Group Cat# 14787‐1‐AP, RRID:AB_2070970), anti‐human RUNX2 at dilution 1:500 (Proteintech Group Cat# 20700‐1‐AP, RRID:AB_2722783), rabbit anti‐human ENPP2 at dilution 1:500 (Thermo Fisher Scientific Cat# PA5‐12478, RRID:AB_2231274), and mouse anti‐human Ankyrin G1 antibody at dilution 1:500 (Innovative Research Cat# 33‐8800, RRID:AB_87179). Next, HRP‐labelled anti‐rabbit IgG antibody was used as a secondary antibody. Complexes of antigen–antibody were visualized with a HRP chemiluminescence detection system (Amersham Biosciences). After detection, membranes were stripped and reprobed for GAPDH. Human CSE recombinant protein (MyBiosource, MBS144930) was used as a control for CSE western blot.

### 2.10 Quantitative real‐time PCR

VIC were cultured in growth media or calcification media supplemented with 20‐μmol·L^−1^ AP72. After 5 days, cells were harvested. Total RNA was isolated using RNAzol STAT‐60 according to the manufacturer's instructions (TEL‐TEST Inc., Friendswood, TX, USA). RNA concentration was measured with NanoDropTM 2000c spectrophotometer (Thermo Scientific Inc., Waltham, MA, USA). Subsequently, cDNA synthesis was performed using a high‐capacity cDNA kit (Applied Biosystems, Foster City, CA, USA). We used real‐time PCR technique for quantification of mRNA levels of ENPP2 and ANK1 (Thermo Fisher Scientific Inc.) and GAPDH (Thermo Fisher Scientific Inc.). TaqMan Universal PCR Master Mix was purchased from Applied Biosystems. Finally, we performed TaqMan quantitative PCR (40 cycles at 95°C for 15 s and 60°C for 1 min) on 96‐well plates with the Bio‐Rad CFX96 (Bio‐Rad Laboratories Inc., Hercules, California, USA) detection system. Results were expressed as mRNA expression normalized to GAPDH.

### 2.11 Intracellular phosphate uptake measurement

The VIC were cultured on 12‐well plates exposed to calcification medium, with or without of phenol red using DMEM supplemented with/without AP72 (20 μmol·L^−1^) for 5 days. Cells were lysed with 0.5% NP40 and 1% Triton‐X100. Whole cell lysate centrifuged at 12,000× *g* for 15 min at 4°C. The supernatant was measured by QuantiChrom quantitative colorimetric phosphate assay kit (BioAssays System) on 96‐well plates at 650 nm. Phosphate uptake was normalized to the protein content of the cells.

### 2.12 Pyrophosphate assay

VIC were cultured in phenol red‐free growth medium (DMEM; Sigma) or calcification medium and supplemented with AP72 (20 μmol·L^−1^). Heart valve tissues (AS *N* = 3; AI *N* = 3) and cells were lysed with EDTA free detergent. Inorganic pyrophosphate (PPi) was measured in the extracellular fluid of the VIC using PPiLight^™^ inorganic pyrophosphate assay (Lonza; LT‐07‐610). The continuous kinetic assay was employed according to the manufacturer's instruction. The luminescence was monitored for 2 hr using Synergy^™^ HTX Multi‐Mode Microplate Reader from BioTek Instruments (USA) with 0.1‐s integrated reading time. The relative luminescence was normalized to the protein content of the cells.

### 2.13 Determination of sulfide level from AS and AI valve tissues with zinc precipitation assay

Sulfide levels were measured with the zinc precipitation method developed by Gilboa‐Garber (1971) and improved by Ang, Konigstorfer, Giles, and Bhatia (2012). The human valves were pulverized under liquid N_2_. Next, the samples were taken up in PBS (pH 7.4), followed by sonication. After that, the sample was centrifuged at 12,000× *g* for 15 min, and the lipid‐free, clear supernatant was collected; 200‐μl sample was mixed with 350 μl 1% zinc acetate and 50 μl 1.5‐mol·L^−1^ sodium hydroxide and incubated for 60 min on a shaker. The incubation step was followed by centrifugation at 2,000× *g* for 5 min to pellet the generated zinc sulfide. The supernatant was then removed, and the pellet was washed with 1 ml of distilled water by vortexing extensively, followed by centrifugation at 2,000× *g* for 5 min. The supernatant was then aspirated off, and the pellet reconstituted with 160 μl of distilled water and mixed with 40 μl of pre‐mixed dye (20 μl of 20‐mmol·L^−1^ dimethyl‐*p*‐phenylenediamine dihydrochloride in 7.2‐mol·L^−1^ hydrochloric acid [HCl] and 20 μl of 30 mmol·L^−1^ iron [III] chloride [FeCl_3_] in 1.2‐mol·L^−1^ HCl). After 10 min, the absorbance of the generated methylene blue (MB) was measured with a spectrophotometer at 667 nm. During the reaction 1‐mol·L^−1^ MB is formed from 1‐mol·L^−1^ sulfide. Thus, the concentration of sulfide is determined using the extinction coefficient (30 200 M^−1^ cm^−1^) of MB. Samples were normalized for protein concentration. Results are presented as μmol·L^−1^ H_2_S generated mg^−1^ protein at 60 min.

### 2.14 CSE and CBS double gene silencing

CSE and CBS genes were silenced, using appropriate siRNAs (Ambion, 4392420; s3710). Briefly, the VIC were cultured on 12‐well plates in antibiotic‐free medium (DMEM, Sigma). At about 70% of confluence, cells were transfected with siRNA against CSE and CBS (Ambion, 4390824; s289). Transfection occurred for 4 hr in minimal serum‐content medium (Opti‐MEM; Gibco). At the end of transfection, 30% FBS containing antibiotic‐free DMEM was added. Next day, cells were washed and treated with AP72 every second day until 5 days. The sequences of the siRNAs were inserted in Data S1.

### 2.15 Pharmacological inhibition of CSE and CBS

VIC were cultured in 12‐well plates in growth medium or calcification medium. Inhibition of CSE, CBS, and http://www.guidetopharmacology.org/GRAC/FamilyDisplayForward?familyId=279#1446 were carried out with the relevant inhibitors, alone or in combination: DL propargylglycine (PPG), amino‐oxyacetic acid (AOAA), or α‐ketoglutaric acid (KGA), AOAA + PPG, AOAA + KGA, and PPG + KGA (each inhibitor at 20 μmol·L^−1^).

### 2.16 Double immunostaining of smooth muscle actin, CSE, and ALP

Fresh valve tissue (AI and AS) samples were fixed in 10% neutral buffered formalin for 2 days and embedded in paraffin wax. Sections (3‐μm) of tissues were deparaffinized, mounted on slides and stained for ALP‐CSE or α smooth muscle actin (α‐SMA; Santa Cruz Biotechnology Cat# sc‐32251, RRID:AB_262054)‐CSE. In case of the calcified sample, the tissue was decalcified. Double immunostaining was performed sequentially with the EnVision FLEX/HRP system. Antibodies were used at a dilution of 1:1,000 in each case. In addition to DAB (brown colour), chromogen VIP was used to highlight the second immunohistochemical reaction in a different colour (dark violet). For double staining experiments, methyl‐green counterstaining was performed.

### 2.17 Immunohistochemistry from mouse heart valves

Briefly, tissues were fixed in formaldehyde for 1 day followed by Tris buffer and embedded in paraffin wax. Subsequently, slides were deparaffinized in xylene and then rehydrated. For immunohistochemistry, slides were subjected to the peroxidase‐blocking reagent. Samples were incubated with the following primary antibodies: anti‐CSE antibody at dilution 1:1,000 (Proteintech Group Cat# 12217‐1‐AP, RRID:AB_2087497) and anti‐SMA antibody at a dilution of 1:1,000 (Santa Cruz Biotechnology Cat# sc‐32251, RRID:AB_262054). Antibody binding was visualized by the Super Sensitive^™^ One Step Polymer‐HRP IHC Detection System. Liquid DAB chromogen (BG‐QD630‐XAKm BioGenex) was added for samples. The intensity and distribution of antibodies expression were assessed by light microscopy (Leica DM2500 microscope, DFC 420 camera, and Leica Application Suite V3 software, Wetzlar, Germany).

### 2.18 LDH cytotoxicity assay

The cytotoxicity of the treatments was assessed by Pierce LDH Cytotoxicity Assay Kit (Thermo Scientific) according to the manufacturer's instructions.

### 2.19 Data and statistical analysis

Data were analysed by GraphPad Prism 5.02 software (GraphPad Software Inc., 7825 Fay Avenue, Suite 230 La Jolla, CA 92037; RRID:SCR_002798). All data are expressed as mean ± SEM. If data groups passed the normality test and equal variance test, we performed Student's *t* test or one way ANOVA followed by Bonferroni post hoc tests as indicated in figure legends. *P* < .05 was considered significant.

### 2.20 Materials

All chemicals were analytical reagent grade or higher and obtained from Sigma‐Aldrich (St Louis, MO, USA). The sulfide donor molecules used in this work—GYY4137 (P‐(4‐methoxyphenyl)‐P‐4‐morpholinylphosphinodithioic acid morpholine salt), AP67 (4‐methoxyphenyl)(pyrrolidin‐1‐yl)phosphinodithioc acid), and AP72 (4‐methoxyphenyl)(piperidin‐1‐yl)phosphinodithioc acid)—were synthesized in‐house (Kulkarni‐Chitnis et al., 2015; L. Li et al., 2008; Whiteman et al., 2015). Sulfide stock solutions were prepared fresh daily in water.

### 2.21 Nomenclature of targets and ligands

Key protein targets and ligands in this article are hyperlinked to corresponding entries in http://www.guidetopharmacology.org, the common portal for data from the IUPHAR/BPS Guide to PHARMACOLOGY (Harding et al., 2018), and are permanently archived in the Concise Guide to PHARMACOLOGY 2017/18 (Alexander, Fabbro et al., 2017; Alexander, Kelly et al., 2017)

## 3 RESULTS

### 3.1 H2S prevents calcification of VIC

**Figure 1:**
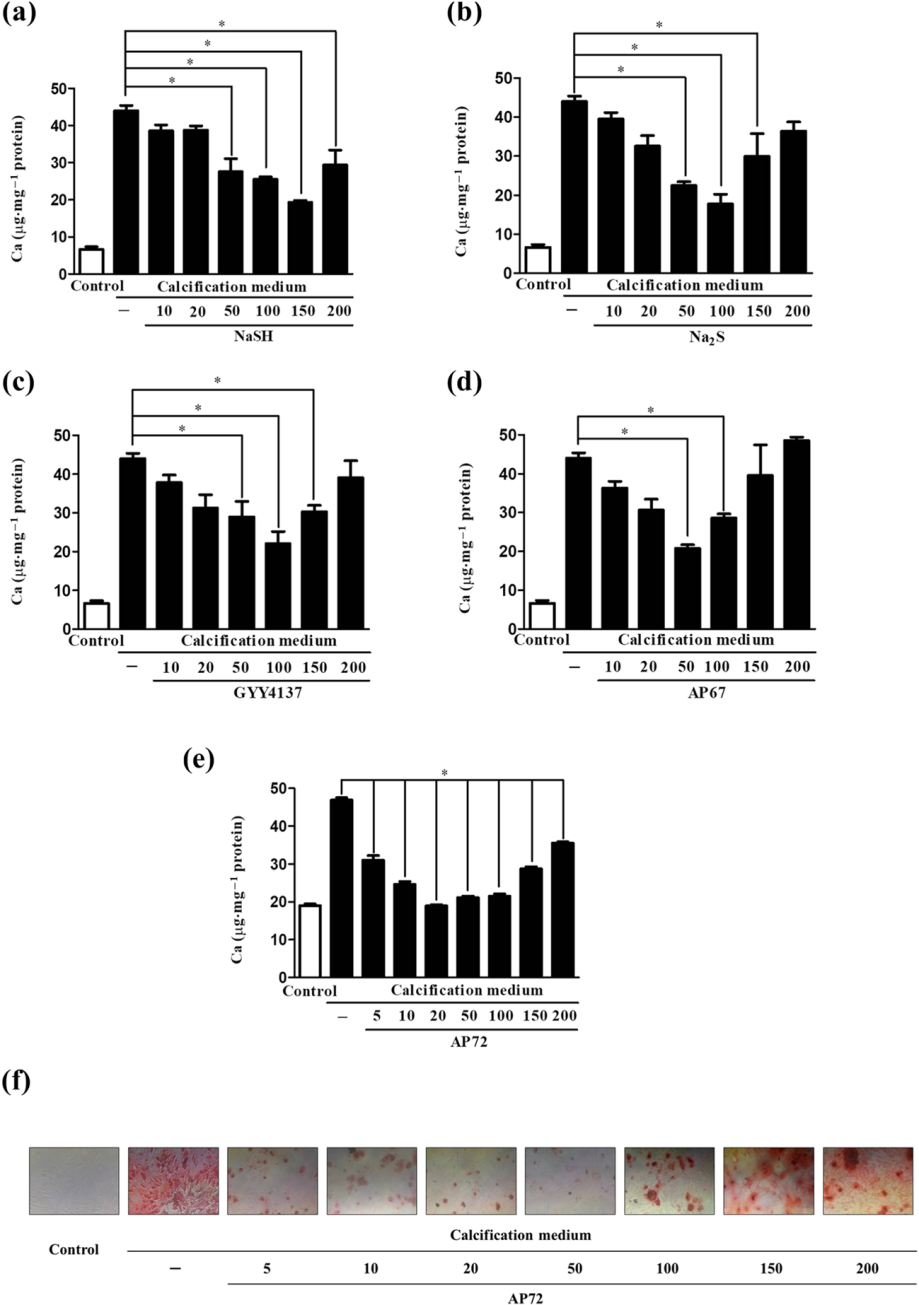
H_2_S donors dose‐dependently inhibit the calcification of VIC. Calcium contents of VIC in growth medium or calcification medium are shown, after 5 days treatment with (a) NaSH, (b) Na_2_S, (c) GYY4137, (d) AP67 (10–200 μmol·L^−1^), or with (e) AP72 (5–200 μmol·L^−1^). (f) Alizarin Red S staining of VIC. Data shown are means ± SEM of five independent experiments. *P < .05, significantly different as indicated

**Figure 2:**
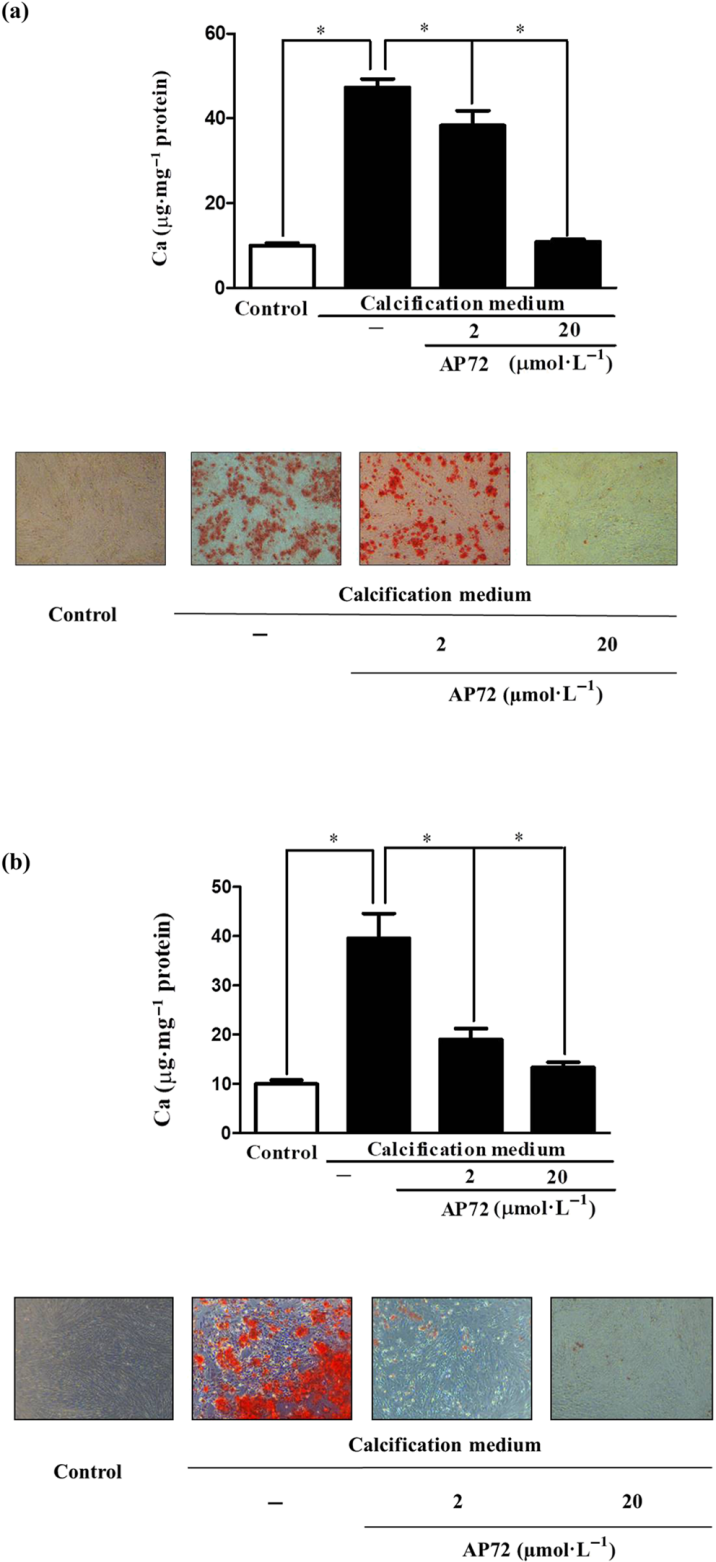
Phenol red impairs anti‐calcification effect of H_2_S. Cultured VIC in (a) phenol red‐containing DMEM (Sigma) or (b) phenol red‐free media were supplemented with AP72 (2 μmol·L^−1^; 20 μmol·L^−1^) for 5 days, and calcium content of the cells was measured and normalized to protein content of the cells. Alizarin Red S staining represents the microscopic image of calcium deposition of extracellular matrix. Data shown are means ± SEM of five independent experiments. *P < .05, significantly different as indicated

We compared the potential of different H_2_S donors for inhibiting calcification of VIC isolated from human aortic valves. Cells were maintained in calcification medium containing 2.5‐mmol·L^−1^ inorganic phosphate and 1.8‐mmol·L^−1^ calcium–chloride. VIC were treated with H_2_S from two sources. One source of H_2_S was the simple sulfide salts NaSH and Na_2_S that instantaneously generates H_2_S via pH‐dependent salt dissociation and the other was the novel slow‐release sulfide donors (AP67 and AP72) and the more commonly used donor GYY4137 (synthesized in‐house). As expected, the transition of VIC into osteoblasts occurred in the calcifying environment, which is reflected by the calcium accumulation in the extracellular matrix (Figure 1) and the increased expression of osteocalcin and ALP (Figure 2). Importantly, all H_2_S donors decreased calcium deposition in a dose‐dependent fashion (Figure 1). NaSH reached the maximum inhibition at 150 μmol·L^−1^ (Figure 1a), Na_2_S and GYY4137 attenuated calcification at 100 μmol·L^−1^ (Figure 1b,c), while AP67 suppressed calcification at 50 μmol·L^−1^ (Figure 1d), compared to calcification medium without H_2_S supplementation. Among the H_2_S donors, AP72 fully prevented calcium deposition in the extracellular matrix of VIC at 20 μmol·L^−1^ concentration (Figure 1e,f). Furthermore, osteocalcin accumulation and expression of ALP in VIC along with calcium deposition were also prevented by AP72 (20 μmol·L^−1^; Figure S3A–C). Similarly, other fast (NaSH and Na_2_S) and slow (GYY4137 and AP67) sulfide‐releasing compounds significantly attenuated the secretion of osteocalcin (Figure S1A). ALP and Alizarin Red S staining showed pronounced osteoblastic transformation of VIC in the calcific environment, and this effect was prevented by AP72 (Figure S3C,D). As demonstrated in Figure S3E, AP72 did not exhibit any cytotoxic effects on VIC at the applied dose. We observed a “U” shape curve in the inhibition of mineralization. The use of H_2_S donors at concentrations in excess of that stated above resulted in a concentration‐dependent decline in protection. From these studies, we then selected the most effective H_2_S donor (AP72) for further investigation to explore the mechanism by which H_2_S regulates the calcification processes.

As phenol is known to capture H_2_S (Huang, Zhang, Zhou, Tao, & Fan, 2017), we tested if AP72 affects calcification at lower concentrations in phenol red‐free medium, compared to calcification medium with phenol red. Without phenol red, AP72 significantly inhibited calcification of VIC at concentration of 2.5 nmol·L^−1^ to 5 μmol·L^−1^ (Figure S1B). Accordingly, osteocalcin accumulation was prevented, and phosphate uptake was also decreased by AP72 in phenol red‐free condition (Figure 2a,b). ALP and Alizarin Red S staining demonstrated the inhibitory effect of AP72 at a concentration of 2 μmol·L^−1^ (Figure 2d,e). As shown in Figure S3, in phenol red‐containing medium, AP72 exhibited inhibitory effect on calcification in VIC at one‐order magnitude higher concentration. There was no cytotoxicity in the VIC cultures due to the sulfide donor compounds, at the most effective concentrations (Figure S1C).

### 3.2 AP72 inhibits the phosphate‐induced nuclear translocation of RUNX2

**Figure 3:**
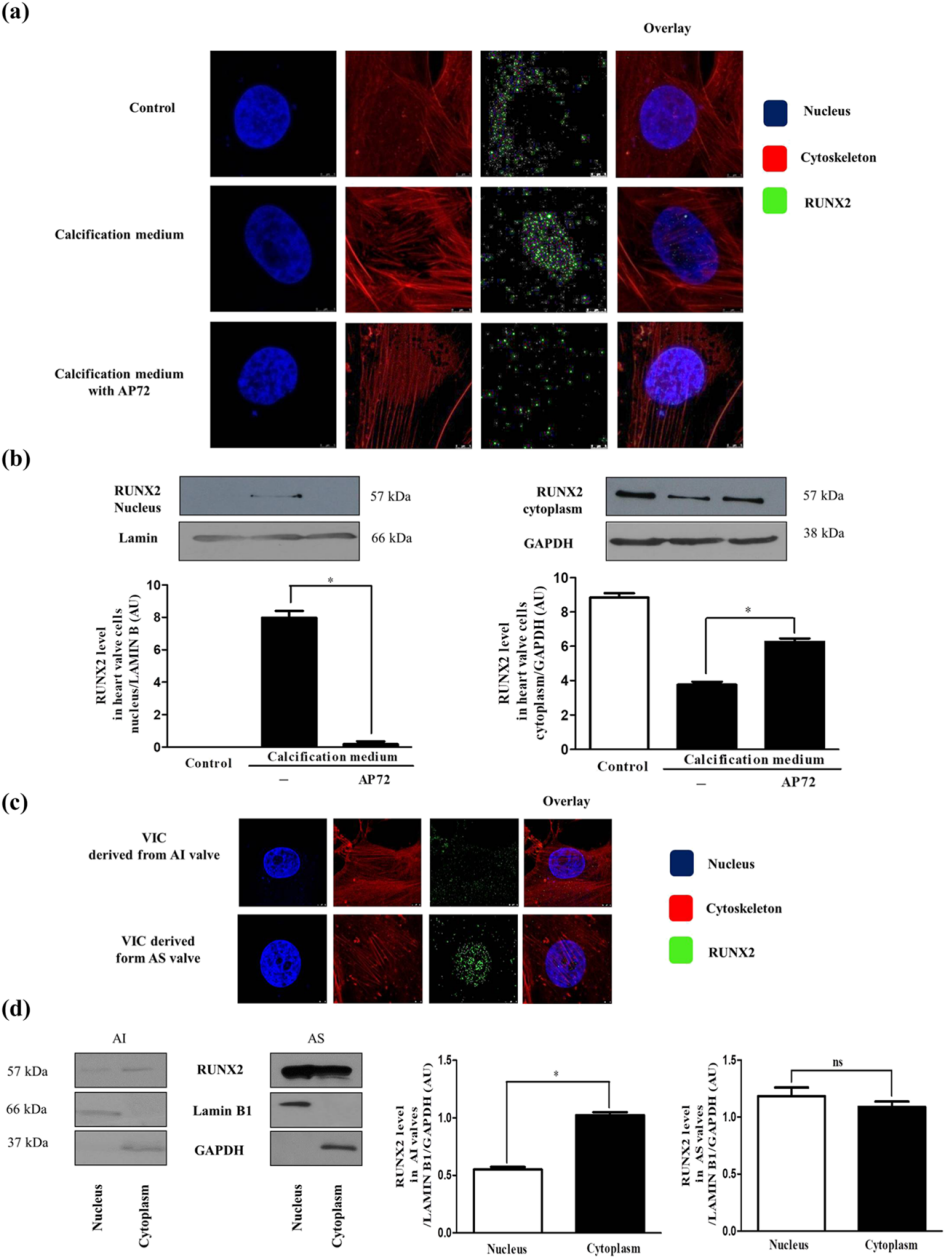
AP72 prevents nuclear translocation of RUNX2. VIC grown on coverslips were exposed to growth medium or calcification medium alone or supplemented with AP72 (20 μmol·L^−1^) for 2 days. (a) Cells were stained for DNA (Hoechst 33258, blue), RUNX2 (green, Alexa Flour 488), and F‐actin (cytoskeleton, red, iFluor 647). Images are obtained employing an immunofluorescence‐confocal STED nanoscope. (b) RUNX2 expression in cytoplasmic and nuclear fraction of VIC. The band intensities are normalized for Lamin B1 in case of nuclear extracts and for GAPDH in case of cytoplasm extracts. (c) RUNX2 localization in AI‐ and AS‐derived VIC samples. Images are obtained employing an immunofluorescence‐confocal STED nanoscope. (d) Protein levels of RUNX2 in AI and AS tissue lysates are shown. Representative staining and protein analysis are shown from five independent experiments. *P < .05, significantly different as indicated; ns, not significant

RUNX2 is a key transcription factor associated with an early osteoblastic differentiation of vascular smooth muscle cells and VIC. We therefore assessed the effects of AP72 treatment on the localization of RUNX2 in VIC cultured in calcification medium. Immunofluorescence staining indicated that RUNX2 was located in the cytoplasm of VIC cultured in growth medium (control medium; Figure 3a; upper panels). Phosphate exposure of VIC triggered the translocation of RUNX2 from the cytoplasm to the nucleus (Figure 3a; middle panels). As demonstrated in Figure 3a (lower panel), AP72 prevented the appearance of RUNX2 in the nucleus of VIC maintained in calcification medium. To support our immunofluorescence observation, cytoplasmic and nuclear fractions of VIC were examined for RUNX2 using western blot analysis. We found that RUNX2 appeared in the nucleus in response to calcification medium (Figure 3b; left panel), while its level was decreased in the cytoplasmic fraction (Figure 3b; right panel). Importantly, AP72 treatment prevented the translocation of RUNX2 into the nucleus of VIC exposed to phosphate (Figure 3b).

Next, we examined the nuclear location of the RUNX2 in VIC derived from human valves with visible calcification (AS) and without calcification (AI). Confocal microscopy and western blot analysis showed that RUNX2 was mainly located in the nucleus of VIC derived from AS human valve tissue, whereas RUNX2 was detected in the cytoplasm of VIC derived from AI tissue (Figure 3c,d). Importantly, exposure of cells to 200 μmol·L^−1^ of AP72 did not restrain the nuclear translocation of RUNX2 from the cytoplasm to the nucleus (Figure S4).

### 3.3 Hydrogen sulfide enhances PPi production

**Figure 4:**
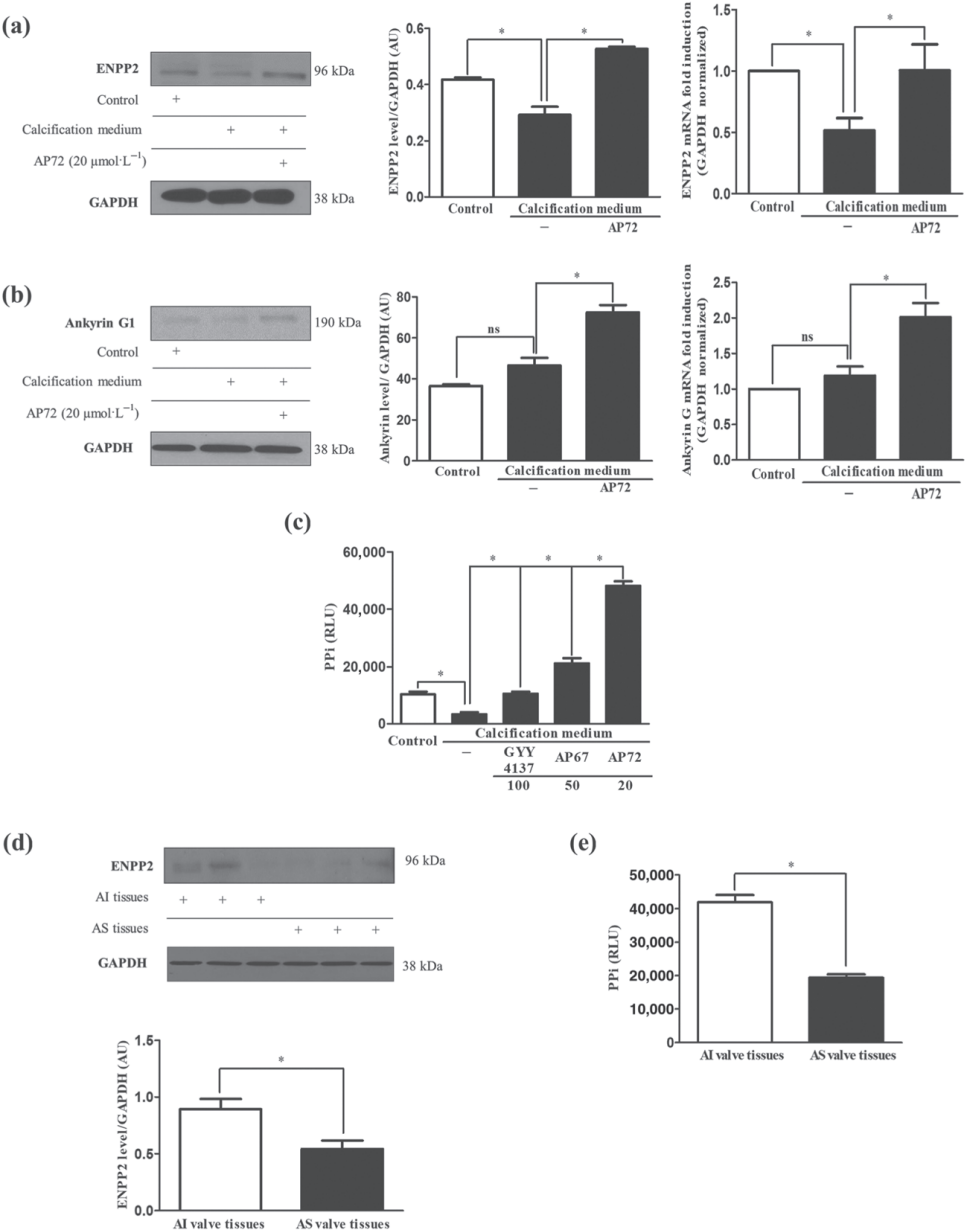
AP72 enhances generation of PPi. VIC were cultured in growth medium or calcification medium alone or supplemented with AP72 (20 μmol·L^−1^) for 5 days. Differences in (a) ENPP2 protein and mRNA levels, (b) ANK1 protein and mRNA levels, (c) pyrophosphate level measured using a PPiLight pyrophosphate detection kit are presented. (d) Representative ENPP2 western blot from AI and AS tissue lysate of heart valves. (e) Pyrophosphate levels of heart valve tissues were measured using a pyrophosphate detection kit. Data shown are means ± SEM of five independent experiments. *P < .05, significantly different as indicated; ns, not significant

PPi is a well‐known anti‐calcification molecule which is regulated by ENPP2 and ANK1. Therefore, we tested the effects of H_2_S on PPi production, through regulating the expression of ENPP2 and ANK1 in VIC. As shown in Figure 4a, expression of ENPP2 was decreased in VIC cultured in calcification medium as compared to control. Treatment of cells with AP72 abolished such an effect. ENPP expression was significantly elevated above the level observed in control cells (Figure 4a). Furthermore, the level of ANK1 protein in VIC maintained under calcifying conditions did not change compared to cells grown in growth medium. In contrast, AP72 induced ANK1 expression at both mRNA and protein levels in VIC (Figure 4b). Accordingly, the amount of PPi generated was decreased in cells grown under calcifying conditions (Figure 4c). In the presence of AP72, PPi generation increased significantly in VIC, when compared with cells cultured in both control growth medium and calcification medium without any treatment (Figure 4c). We also tested GYY4137 and AP67 for their effects on the PPi levels in VIC. These donors enhanced the PPi level less effectively than AP72 (Figure 4c). The fast sulfide‐releasing molecules (NaSH and Na_2_S) were able to enhance the PPi level only to the baseline (Figure S5). Moreover, our measurements in human heart valve tissue samples indicated significantly lower ENPP2 protein levels and lower PPi content in AS valve specimens than those in samples from AI valves (Figure 4d,e).

### 3.4 Attenuated CSE and CBS expression promotes VIC calcification

**Figure 5:**
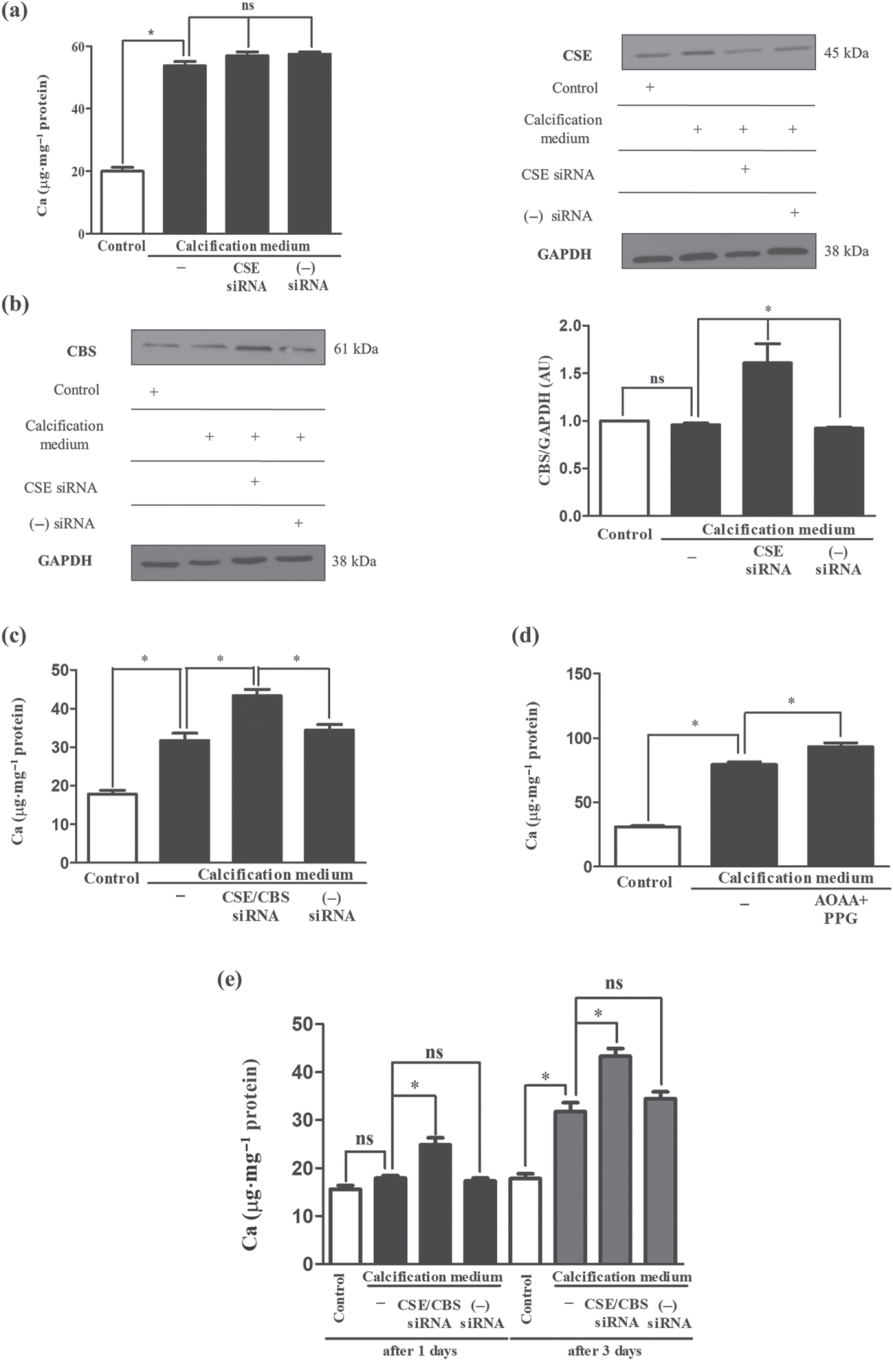
Simultaneous silencing or pharmacological inhibition of CSE and CBS increases calcification of VIC. VIC were cultured in growth medium or calcification medium. Cystathionine‐γ‐lyase (CSE) and CSE/CBS (cystathionine‐β‐synthase) double gene silencing using siRNA was performed. (a) Calcium content, (b) CSE and CBS levels of VIC after silencing of CSE are shown. Panel (c) shows the calcium content of CSE/CBS double silenced samples. Panel (d) shows the calcium contents of pharmacological inhibition of CSE and CBS. (e) Calcium depositions of CSE/CBS double silenced VIC were shown. Data shown are means ± SEM of five independent experiments. *P < .05, significantly different as indicated; ns, not significant

To investigate potential anti‐calcification effects of endogenously produced H_2_S we first silenced CSE production in VIC using siRNA. We found that CSE silencing (Figure 5a; right panel) did not significantly enhance the calcium accumulation under calcifying conditions (Figure 5a; left panel). Intriguingly, we observed that CSE silencing increased the expression of CBS, which is another pyridoxal 5′‐phosphate (PLP or vitamin B6) dependent transsulfuration enzyme involved in endogenous sulfide production (Figure 5b). Thus, we silenced both CSE and CBS to test whether calcium deposition in extracellular matrix was affected. We observed an increase in mineralization after CSE and CBS were concomitantly silenced in VIC (Figure 5c) suggesting that transsulfuration pathways are likely to control calcification. Next, we performed experiments with pharmacological inhibitors of CSE (PPG) together with CBS (AOAA), in VIC under calcifying conditions. We found that pharmacological inhibition of CSE with CBS increased the amount of extracellular calcium deposition (Figure 5d). Furthermore, we tested all three synthetic inhibitors for CSE (PPG), CBS (AOAA), and 3‐MST (KGA) alone or in combination. Calcium depositions of CSE/CBS double silenced VIC shown inhibition of CSE and CBS altered calcification in VIC (Figure S6A). Furthermore, AOAA + PPG; AOAA + KGA, and PPG + KGA pharmacological inhibitors significantly reduced the H_2_S production in VIC (Figure S6B). To our surprise, nuclear translocation of RUNX2 was not influenced by double silencing of CSE and CBS in calcification medium (Figure S8). Furthermore, we monitored the progression of calcification in extracellular matrix on the first and third days. On the first day, we did not find significant alteration in the calcium content of VIC maintained in calcifying condition compared to control. In contrast, we observed a significantly increased extracellular calcium content in VIC silenced with CSE/CBS siRNA (Figure 5e). Mineralization was more robust in the double silenced VIC by day three (Figure 5e). As shown in Figure S7B, production of H_2_S was lowered in calcifying conditions, compared to cells cultured in growth media and that was further decreased by double silencing for CSE and CBS. Finally, we examined the expression of 3‐MST in VIC treated with CSE/CBS siRNA. We found that siRNA specific to CSE and CBS decreased 3‐MST protein level in VIC (Figure S7C). Western blots of CSE and CBS of double silenced cells are shown in Figure S8.

### 3.5 Hydrogen sulfide inhibits phosphate uptake through affecting the functions of phosphate channels

**Figure 6:**
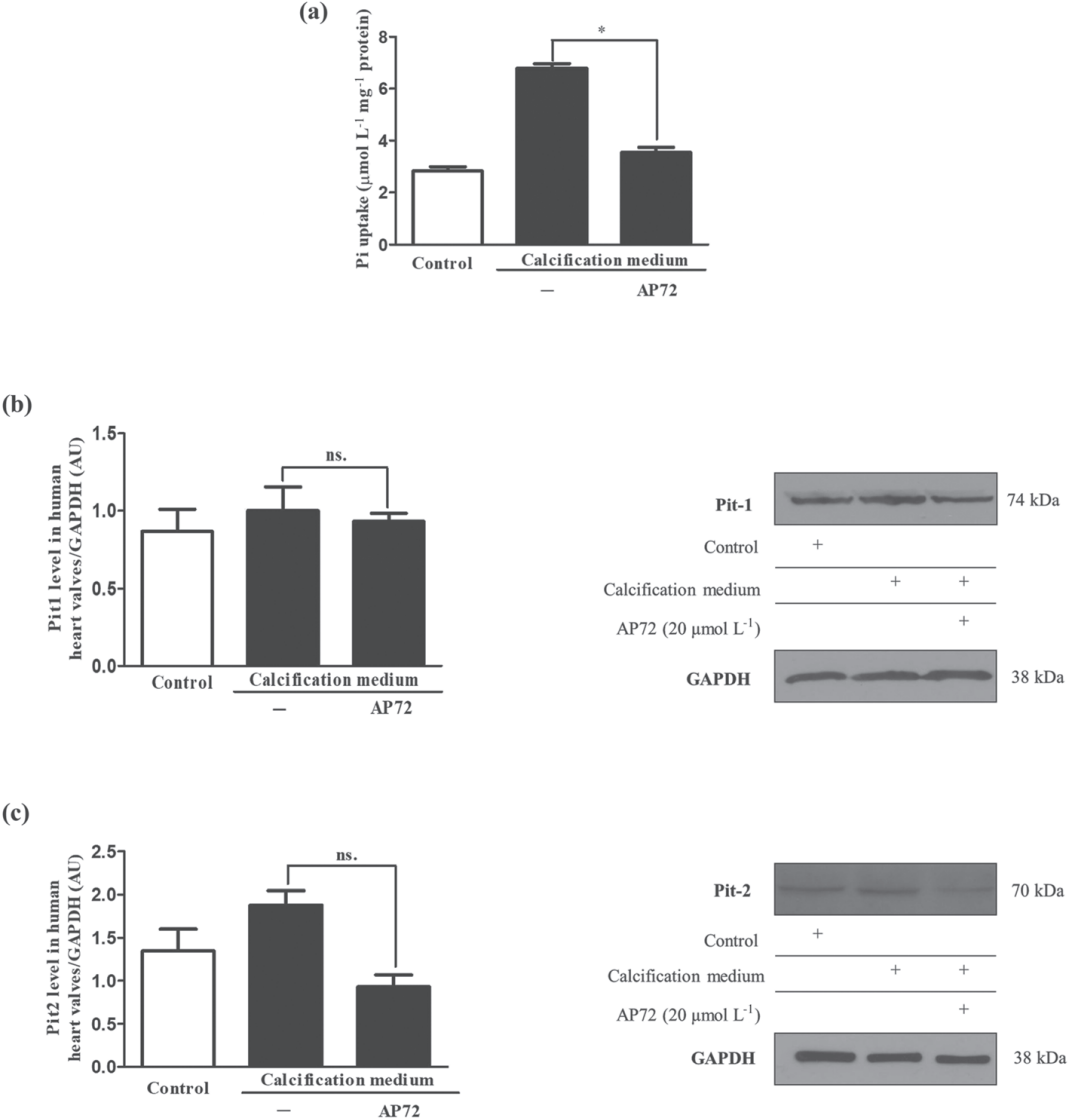
Hydrogen sulfide donors leads to inhibition of phosphate transport. Cells were cultured in a normal or calcific environment exposed to AP72 (20 μmol·L^−1^) for 5 days. (a) Phosphate content was determined using QuantiChrom quantitative colorimetric assay. (b) Pit1 and (c) Pit2 western blotting was performed and normalized to GAPDH. Data shown are means ± SEM of five independent experiments. *P < .05, significantly different as indicated; ns, not significant

As cellular phosphate uptake is a key event in the mineralization process, we therefore next measured intracellular phosphate levels in VIC and observed a significant elevation in cells cultured in calcification medium, compared to cells kept in control medium (Figure 6a). Exposure of VIC to AP72 diminished this increase in phosphate content to the level observed in control cells (Figure 6a). In order to explain the inhibition of phosphate uptake, we measured the expression of phosphate channels (Pit1 and Pit2) in VIC maintained in calcification medium, with or without AP72. We found that AP72 did not affect the expression of Pit1 and Pit2 channels (Figure 6b,c). Therefore, we hypothesize that sulfide‐induced post‐translational modification of these channels might affect phosphate uptake. Measurements to support or disprove this hypothesis are underway.

### 3.6 Expression of CSE and generation of H2SH2S in human aortic valves

**Figure 7:**
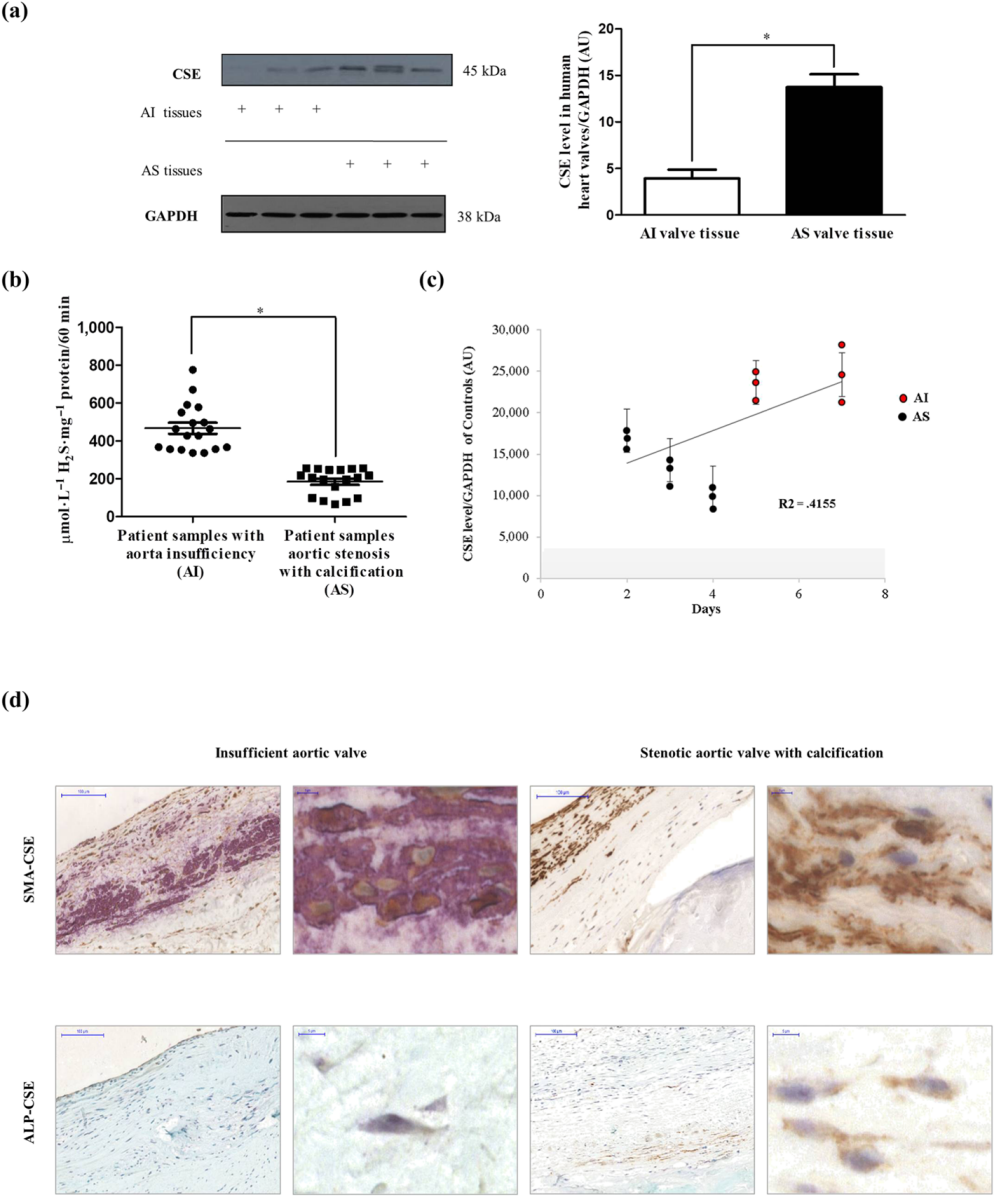
Expression of CSE in human aortic valves. (a) Western blot of CSE protein from AS valve (*N* = 12) lysates and AI valve (*N* = 9) lysates (left panel) were performed. Representative CSE protein levels of three AS and three AI valves lysates were shown. Relative CSE levels were assessed by densitometry analyses of band intensities of CSE western blots normalized to GAPDH (panel right). (b) Zn^2+^ precipitated sulfide content under alkaline conditions was measured in AI (*N* = 18) and AS (*N* = 18) valve tissues normalized to protein content of the individual samples. (c) VIC derived from different AI (*N* = 6) and AS (*N* = 9) patients were cultured in calcification medium. CSE expression was measured after the initiation of calcification. Results were normalized to GAPDH of the samples. (d) Double immunohistochemistry of CSE‐SMA and CSE‐ALP was shown with two different magnifications (×400 magnification and ×1,000 magnification). Data shown are means ± SEM of N experiments. *P < .05, significantly different as indicated

CSE is one of the main endogenous hydrogen sulfide producing proteins in the human body. Using western blot analyses, we investigated the expression of CSE in tissue lysates of human AS and AI valves. We found higher expression of CSE in AS valves with massive calcification, compared with the expression in AI valves known to lack calcification (Figure 7a). In contrast, sulfide levels that can be precipitated by Zn^2+^ under alkaline conditions from tissue lysates of valves were markedly and significantly lower in calcified AS specimens compared to not calcified AI specimens (Figure 7b). Next, we performed dual immunohistochemistry analyses (CSE‐SMA and CSE‐ALP) on human AI and AS valves to localize CSE. As shown in Figure 7, less SMA+ and more CSE+ cells were present in calcified AS tissue than in AI tissue (upper panels). ALP‐CSE double staining revealed the appearance of ALP+ cells expressing high levels of CSE protein in AS valve samples, while ALP+ cells were not detected in AI valve (Figure 7c; lower panels).

The potential of osteoblastic differentiation was dependent upon the origin of VIC. Under the same calcifying conditions, VIC derived from AS exhibited earlier mineralization than AI. The higher CSE level found in AI was accompanied by delayed calcification (Figure 7c). Human CSE recombinant protein was used as a control for the CSE western blot (Figure S8B).

### 3.7 H2S prevents valvular calcification in apolipoprotein E deficient mice

**Figure 8:**
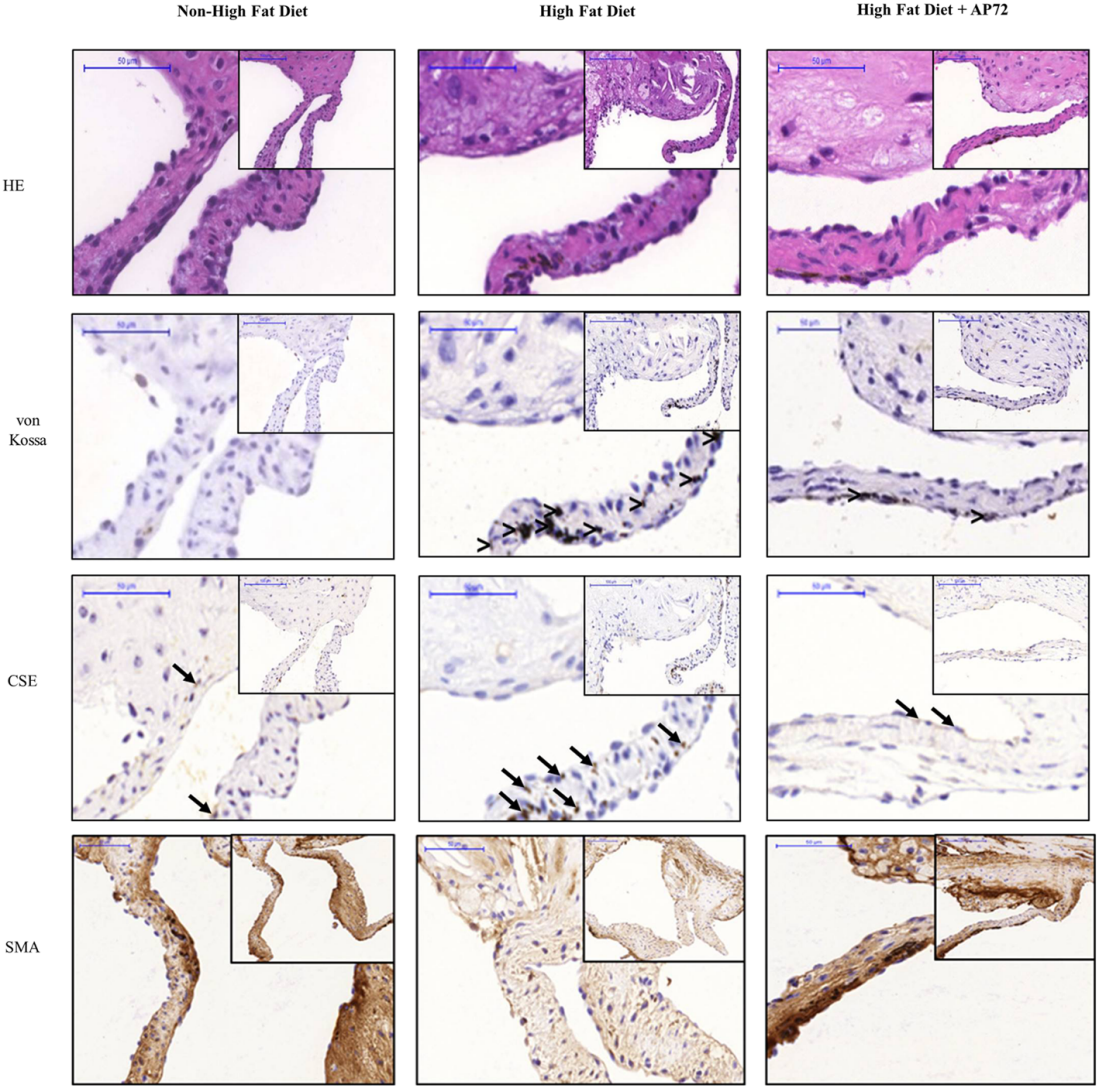
Hydrogen sulfide inhibits valvular calcification in ApoE^−/−^ knockout mice. Haematoxylin and eosin (upper panels), von Kossa (middle panels), CSE (second middle panels), and α smooth muscle actin (α‐SMA; lower panels) staining was performed on aortic valves of mice fed a normal diet (first column; *N* = 5), on a high‐fat diet (second column, *N* = 9); and on a high‐fat diet treated with AP72 (third column; *N* = 5). Comparison (>) designates the calcified regions, and arrow indicates CSE positive cells

As demonstrated by von Kossa staining (Figure 8; middle panels), calcific nodules appeared in aortic heart valves of ApoE^−/−^ mice, on a high‐fat diet (second column; middle panel) as opposed to ApoE^−/−^ mice on standard chow diet, where no valvular calcification was observed (first column; middle panel). ApoE^−/−^ mice on a high‐fat diet exhibited an expansion of extracellular matrix in aortic valve tissue, compared to samples from those that received standard diet (upper panels). To demonstrate the benefit of H_2_S in vivo, we administered AP72 intraperitoneally (266‐μmol·kg^−1^ body weight) and assessed valvular calcification in ApoE^−/−^ mice on a high‐fat diet. AP72 significantly inhibited the development of calcific nodules in aortic valves (third column; middle panels) and decreased the expansion of extracellular matrix (third column; upper panels). As observed in samples from human aortic stenosis, the amount of CSE+ cells in the calcified aortic valves of mice fed with the high‐fat diet was increased as compared to the aortic valves of mice on a regular diet (lower panels). Consequently, the administration of exogenous H_2_S lowered the total measured expression of CSE in aortic valves of mice fed with a high‐fat diet (third column; lower panels). α‐SMA staining demonstrated that AP72 maintained the phenotype of VIC in the aortic valve (Figure 8).

## 4 DISCUSSION

CAVD is the most common valvular heart disease, found mostly in patients with CKD and in the elderly (Freeman & Otto, 2005). With increasing life expectancy, the prevalence of CAVD is expected to rise (Freeman & Otto, 2005; Mohler, Adam, McClelland, Graham, & Hathaway, 1997; O'Brien et al., 1995). More recently it has been proposed that therapies targeting the molecular processes of aortic valve calcification (Yutzey et al., 2014) could increase the durability of surgically implanted and transcatheter bioprosthetic valves (Leopold, 2012).

Increased phosphate levels are a significant risk factor for CAVD and have been demonstrated in many studies to act as a key regulator of vascular calcification (Adeney et al., 2009; Giachelli, 2009; Hruska, Mathew, Lund, Qiu, & Pratt, 2008). It provokes calcification of vascular cells in a process mediated by sodium‐dependent phosphate co‐transporters (Pit1 and Pit2) which facilitate the entry of phosphate into the cells (Zarjou et al., 2009). This induces osteoblastic transition of vascular smooth muscle cells via a process that is accompanied by translocation of RUNX2 from the cytosol into the nucleus required for osteoblast differentiation, bone matrix gene expression, and, consequently, bone mineralization (Komori, 2006; Zarjou et al., 2009). There is also induction of ALP, an important enzyme in early osteogenesis and of osteocalcin, a major non‐collagenous protein found in bone matrix that is demonstrated to regulate mineralization (Zarjou et al., 2009).

We have earlier demonstrated that CSE expression was elevated in human atheroma, derived from the carotid artery, with lipid accumulation without calcification (Potor et al., 2018). In the present study, we found that CSE expression was higher in human AS valves known to be associated with calcification, compared with that in AI valves lacking calcification (Figure 5a). Furthermore, ALP+ cells were shown to exhibit CSE positivity (Figure 7a) in AS valves. Importantly, the generation of H_2_S was decreased in AS heart valve tissue (Figure 7b) compared with that in AI valves, suggesting that loss of CSE activity and/or H_2_S bioavailability might aggravate the progression of mineralization of VIC. To investigate whether endogenous H_2_S production had an anti‐calcification effect, we silenced CSE in VIC isolated from human aortic valves without calcification then cultured the cells in high phosphate containing medium. Based on the observed protecting roles of sulfide administration, we expected that CSE silencing would enhance VIC calcification. In contrast, there was no increase in the calcium deposition after silencing CSE expression (Figure 5a). The interaction between CSE/CBS expression was reported by Nandi and Mishra (2017), demonstrating that CBS deficiency up‐regulates CSE protein levels. Therefore, we investigated the expression of CBS in CSE silenced VIC and found that CSE silencing resulted in an increased CBS protein expression in VIC (Figure 5b). We therefore silenced both CSE and CBS, which further increased calcium accumulation in the extracellular matrix of VIC cultured in high phosphate containing medium (Figure 5c). Furthermore, when we followed the progression of calcification, we found that CSE+CBS silenced VIC underwent calcification earlier compared to cells maintained in calcification medium without siRNA (Figure 5e). Treatment of VIC with a synthetic CSE inhibitor (PPG) together with a CBS inhibitor (AOAA) also enhanced the extracellular calcium deposition (Figure 5d). The protective function of CSE was also supported by the finding that lower expression of CSE found in AS, compared with AI, was associated with higher calcification rate of VIC (Figure 7c). These results are consistent with the notion that there is impaired H_2_S bioavailability and/or defective endogenous H_2_S production in CAVD. Hence, strategies which overcome this loss of H_2_S, such as H_2_S donor compounds, represent a novel strategy for preventing, delaying, and/or reversing mineralization of VIC in CAVD susceptible individuals. However, it has to be noted that CSE and CBS are transsulfuration enzymes working in concert with http://www.guidetopharmacology.org/GRAC/LigandDisplayForward?ligandId=4782 production from homocysteine, via their canonical activities. The metabolic pathways of cysteine (also including the oxidative catabolism as well as the reverse transsulfuration pathways, which can produce H_2_S) are highly orchestrated and fine‐tuned to—among other reasons—keep steady‐state cysteine levels in a narrow concentration range. The homeostatic effects of sulfite and hydrogen sulfide are critical for cysteine catabolism. Therefore, the CSE/CBS double knockdown cell line is expected to not only produce less endogenous H_2_S but also exhibit impaired cysteine formation. The relative contributions of these processes need further investigations.

To gain insight into the contribution of endogenous sulfide production to prevent mineralization of VIC, we employed several H_2_S generating molecules. For example, we used NaSH and Na_2_S which instantly generate H_2_S in aqueous solution as well as the slow sulfide release donor molecules (GYY4137, AP67, and AP72). It is important to note that the concentrations of donors do not represent the total amount of released sulfide, and all slow donors have different sulfide‐releasing potential. In addition, it is increasingly recognized that slow‐releasing H_2_S donors are likely to more closely mimic the effects of the endogenous H_2_S buffer system, because of their slow generation of low sulfide levels (Nagy et al., 2014; Whiteman et al., 2015). In particular, we have shown that slow‐releasing H_2_S molecules such as AP72 (Nagy et al., 2014) exhibited a greater inhibition of the calcification, compared with GYY4137, possibly because the rate of H_2_S release from AP72 is faster than that of GYY4137, a poorly efficient H_2_S donor (Whiteman et al., 2015). It is important to note that phenols are capable of absorbing a high amount of H_2_S from liquids (Huang et al., 2017). Accordingly, in our experiments, AP72 inhibited VIC mineralization at one‐order magnitude lower concentration in phenol red‐free media. Additionally, phosphate uptake of the cells was elevated in the process of calcification followed by nuclear translocation of the RUNX2 from the cytoplasm to the nucleus. Recently, Sun et al. previously demonstrated the importance of RUNX2 in bone and in arterial calcification. They showed that RUNX2 was important in regulating the expression of many factors, including RANKL, which is the key protein in the interplay between venous smooth muscle cells and macrophages and contributes to the pathogenesis of vascular calcification and atherosclerosis (Lin, Chen, Leaf, Speer, & Giachelli, 2015; Sun et al., 2012). Inhibiting phosphate uptake (Figure 6a)—most likely by post‐translational modification of Pit channels—H_2_S attenuated RUNX2 translocation and thereby prevents the appearance of the osteoblastic phenotype (Figure 3), as reflected by ALP, osteocalcin protein expression, and the development of the hydroxyapatite crystals (Figure 2).

Plasma PPi acts as an endogenous inhibitor of vascular calcification, and levels of PPi are reduced in end‐stage renal disease and inversely correlate with arterial calcification (Lomashvili, Narisawa, Millan, & O'Neill, 2014). We therefore asked whether H_2_S could affect the regulation of PPi generation, an observation that could provide a new pathway to inhibit VIC calcification in CAVD. Indeed, in human calcified valves of AS, ENPP2 protein and PPi levels were lower compared to AI valve tissue (Figure 4a,c,d), and we demonstrated that the expression of ENPP2 is markedly up‐regulated by AP72 (Figure 4a) resulting in an increased production of PPi in VIC (Figure 4c). Furthermore, AP72 treatment increased the expression of ANK1 channels which contributed to the extracellular localization of PPi from the cytoplasm (Figure 4b). Taken together, the regulation of PPI generation by H_2_S represents a novel additional mechanism to control calcification.

The ApoE^−/−^ mouse is the most widely studied animal model for atherosclerosis (Massy et al., 2005; Rattazzi et al., 2005). We showed that calcification occurred in the aorta and aortic valves after a high‐fat diet with the most pronounced calcification seen in the aortic arch. In our current study, the development of mineralization in aortic valves of ApoE^−/−^ mice on a high‐fat diet was strongly associated with the increase of extracellular matrix and the formation of hydroxyapatite nodules, and the expansion of extracellular matrix in the heart valves was prevented by AP72. We also observed that similar to human aortic stenosis, the expression of CSE in VIC was increased in mice on a high‐fat diet. Several triggers were previously identified by our group for inducing CSE expression in resident cells of atherosclerotic lesions including plaque lipids, oxidized LDL, TNF‐α, and IL‐1β (Potor et al., 2018). Therefore, the induction of CSE might represent an adaptive cellular response to lipid deposition and inflammation, serving as a clinical biomarker of CAVD. In the process of the calcification, vascular smooth muscle cells were shown to transform into osteoblastic‐like cells and lose their SMA (Johnson, Leopold, & Loscalzo, 2006). In our experiments, immunostaining of the aortic valves of ApoE^−/−^ mice demonstrated that AP72 treatment preserved α‐SMA (Figure 7, lower panels). In keeping with our hypothesis that CAVD is a condition of perturbed H_2_S bioavailability, we found a deficiency in the generation of H_2_S in human AS valves in spite of the elevated expression of CSE. This contradiction can be resolved by the observations of Bibli et al., (2019), who found a markedly decreased H_2_S production after phosphorylation of Ser^377^ of CSE, resulting in enzyme inactivation. As such, therapeutic supplementation of H_2_S using H_2_S donor molecules such as AP72 or other novel compounds that are currently under preclinical or clinical investigation by us and others may offer a novel approach to prevent valvular calcification in CAVD and related conditions.

The pKa values of protein cysteines and their nucleophilicities are mainly dependent on charged states, orientations, inductive effects of neighbouring functional groups, and solvent accessibility of the thiol group (Nagy, 2013). Unfortunately, the accurate crystal structures of Pit1 and RUNX2 are still not known, which makes it virtually impossible to appropriately predict reactive cysteine residues on these proteins, which might be persulfidated with a functional effect on their activities. However, persulfidation of RUNX2 on Cys^123^ and Cys^132^ by CSE‐produced endogenous sulfide enhanced its transactivation (Zheng et al., 2017). This effect is in contrast to our observations that endogenously produced or exogenously administered sulfide inhibited nuclear translocation of RUNX2, suggesting that the anti‐calcification effects of H_2_S are more likely due to the inhibition of phosphate uptake, and the inhibition of RUNX2 translocation in our model is likely to be a secondary effect of this, as we proposed above.

In our study, we identify three separate mechanisms for the anti‐calcification effects of H_2_S (a) inhibiting phosphate uptake, (b) preventing nuclear translocation of RUNX2, and (c) increasing pyrophosphate level. Although all these pathways are important and related to each other, some caveats are acknowledged. AP72 exhibits slight but significant anti‐calcification action at 200 μmol·L^−1^, although it did not affect nuclear translocation of RUNX2 indicating the importance of pyrophosphate for preventing the formation of mineralized nodules in extracellular matrix.

In conclusion, our study has provided evidence that endogenous H_2_S limits calcification of VIC. Pharmacologically generated H_2_S, derived from novel H_2_S‐releasing molecules, may have the potential to control calcification in heart valves and osteoblastic differentiation of VIC.

## CONFLICT OF INTEREST

The authors declare no conflicts of interest. M.W. has patent applications for the therapeutic use of slow‐release hydrogen sulfide donor molecules.

## AUTHOR CONTRIBUTIONS

K.É.S., L.P., M.O., Z.H., R.T., M.W., and J.B. did the conception or design of the work. K.É.S., L.P., M.O., and I.F. collected the data. K.É.S., L.P., M.O., G.M., Z.H., and J.B. analysed and interpreted the data. K.É.S., L.P., M.O., G.M., Z.H., M.W., and J.B. drafted the data. L.P., M.O., P.N., G.M., A.Z., A.A., N.P., M.W., G.B., and J.B. critically revised the article. K.É.S., L.P., P.N., G.M., A.Z., A.A., Z.H., M.W., and J.B did the final approval of the version to be published. T.S., K.É.S., L.P., G.M., Z.H. collected the specimen.

## DECLARATION OF TRANSPARENCY AND SCIENTIFIC RIGOUR

This Declaration acknowledges that this paper adheres to the principles for transparent reporting and scientific rigour of preclinical research as stated in the *BJP* guidelines for https://bpspubs.onlinelibrary.wiley.com/doi/full/10.1111/bph.14207, https://bpspubs.onlinelibrary.wiley.com/doi/full/10.1111/bph.14208, and https://bpspubs.onlinelibrary.wiley.com/doi/abs/10.1111/bph.14206, and as recommended by funding agencies, publishers and other organisations engaged with supporting research.

## Supporting information

Figure S1. Osteocalcin levels, calcium deposition and cytotoxicity in VIC
Figure S2. In phenol red‐free condition AP72 inhibits valvular calcification at 2 μmol/L
Figure S3. Inhibition of valvular calcification by AP72
Figure S4. RUNX2 nuclear translocation in calcifying condition in the presence of 20 and 200 μmol/L of AP72
Figure S5. Changes of PPi levels by fast sulfide donor treatment on VIC
Figure S6. Inhibition of H2S production by pharmacological inhibitors in VIC
Figure S7. CSE/CBS silencing enhanced the progression of calcification and decreased the expression of 3‐MST
Figure S8. CSE and CBS expression of the double silenced VIC
Data S1. Sequences of the siRNA
