# Supplementary material for: Hydrogen sulfide inhibits calcification of heart valves; implications for calcific aortic valve disease
Source: Br J Pharmacol. 2019 Apr 24;177(4):793–809. doi: 10.1111/bph.14691 (PMC7024713; doi:10.1111/bph.14691)
Supplement: Supplementary file 1 — Figure S1. Osteocalcin levels, calcium deposition and cytotoxicity in VIC Figure S2. In phenol red‐free condition AP72 inhibits valvular calcification at 2 μmol/L Figure S3. Inhibition of valvular calcification by AP72 Figure S4. RUNX2 nuclear translocation in calcifying condition in the presence of 20 and 200 μmol/L of AP72 Figure S5. Changes of PPi levels by fast sulfide donor treatment on VIC Figure S6. Inhibition of H2S production by pharmacological inhibitors in VIC Figure S7. CSE/CBS silencing enhanced the progression of calcification and decreased the expression of 3‐MST Figure S8. CSE and CBS expression of the double silenced VIC Data S1. Sequences of the siRNA [file BPH-177-793-s001.pdf]

A

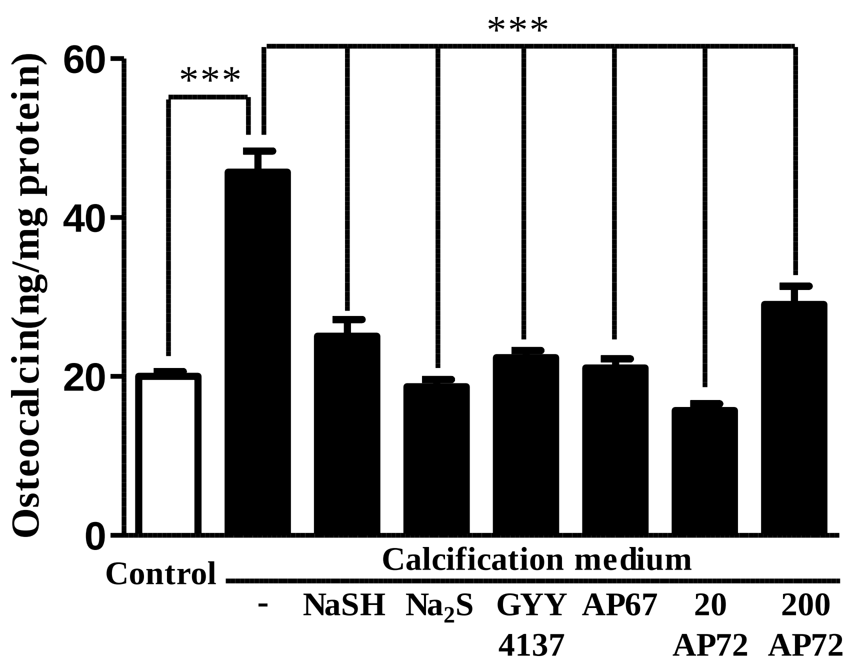

B

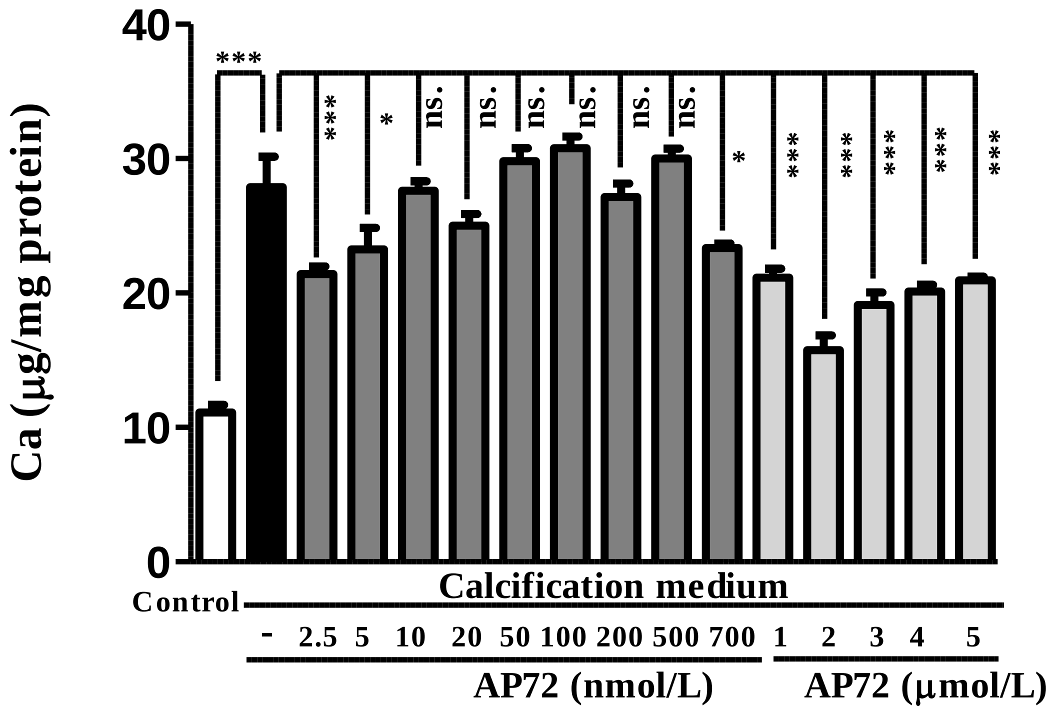

C

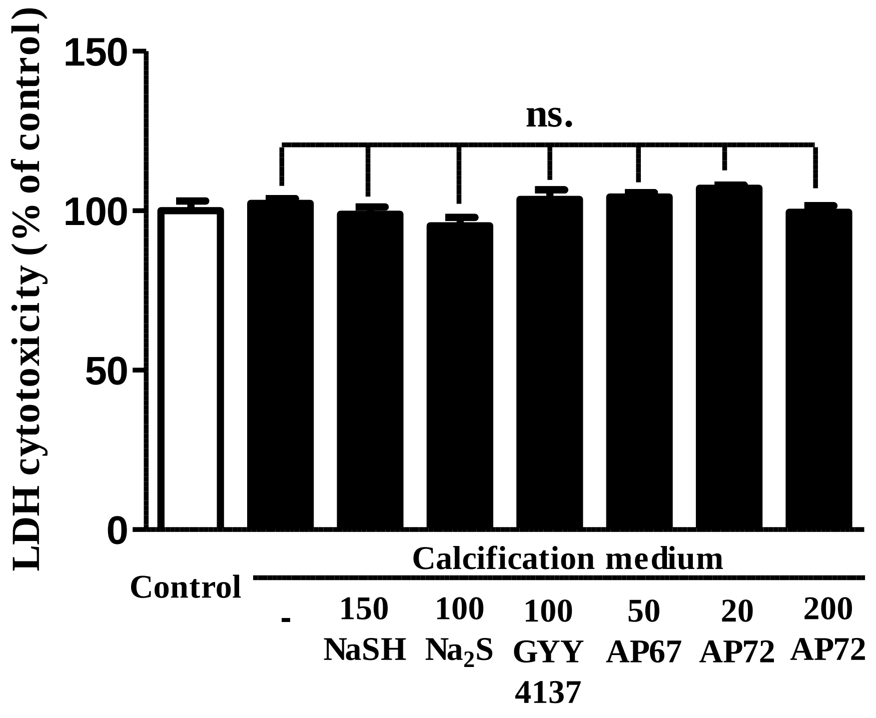

Supplementary Figure 1

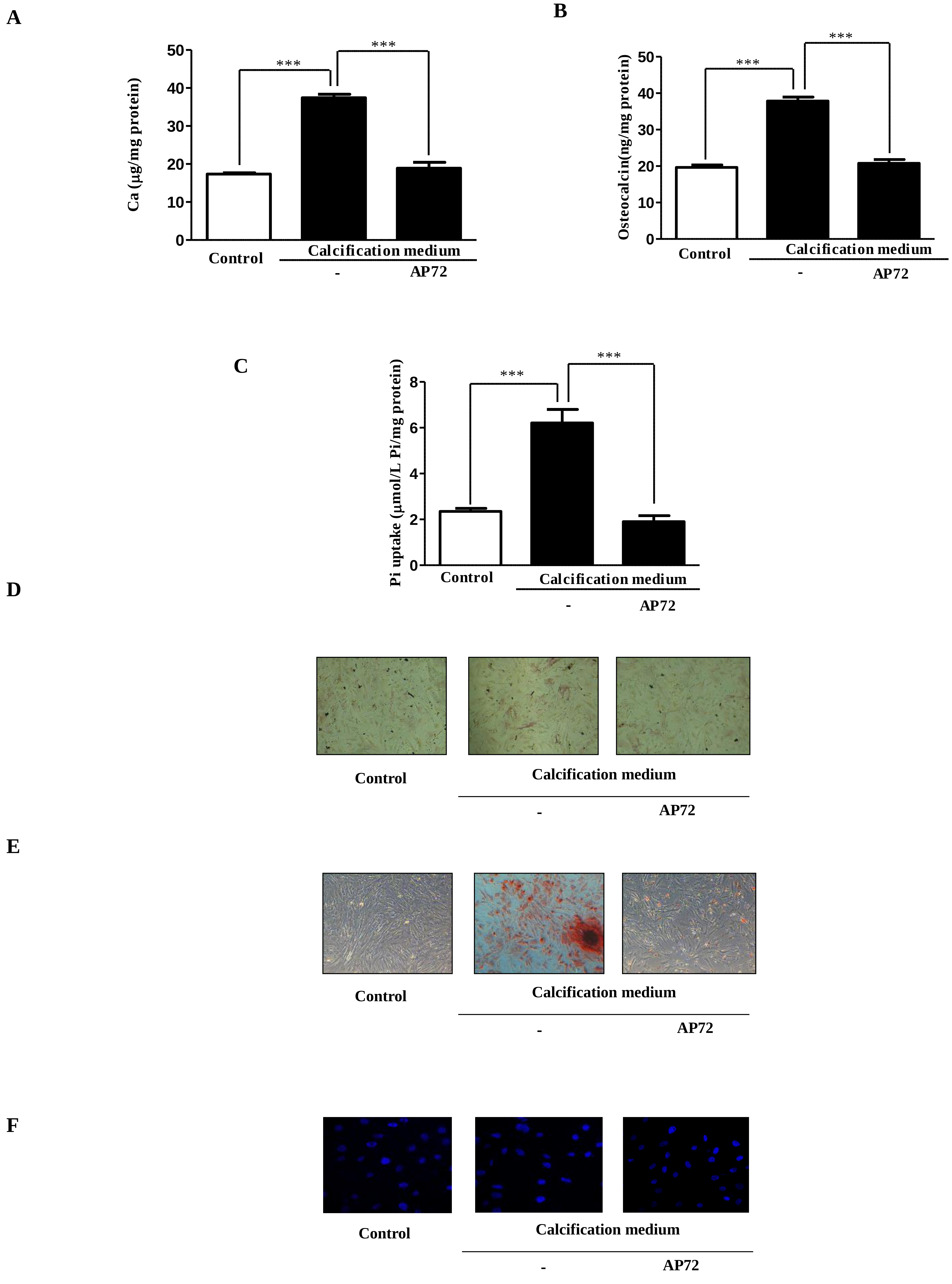

Supplementary Figure 2

A

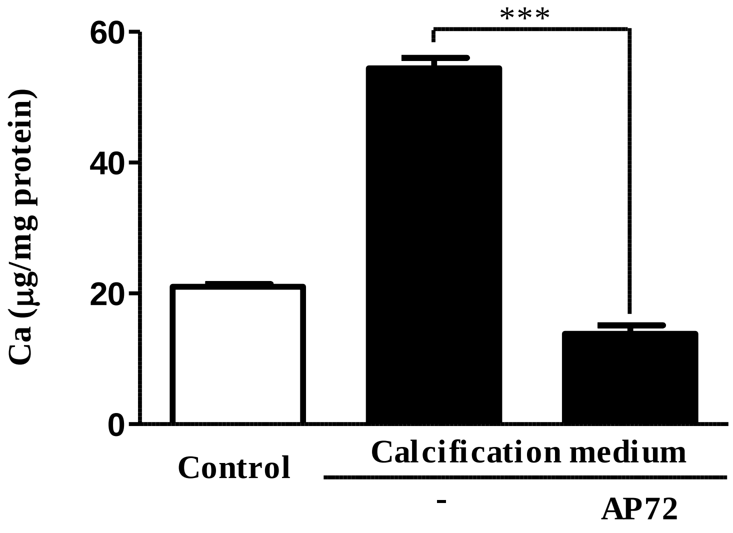

B

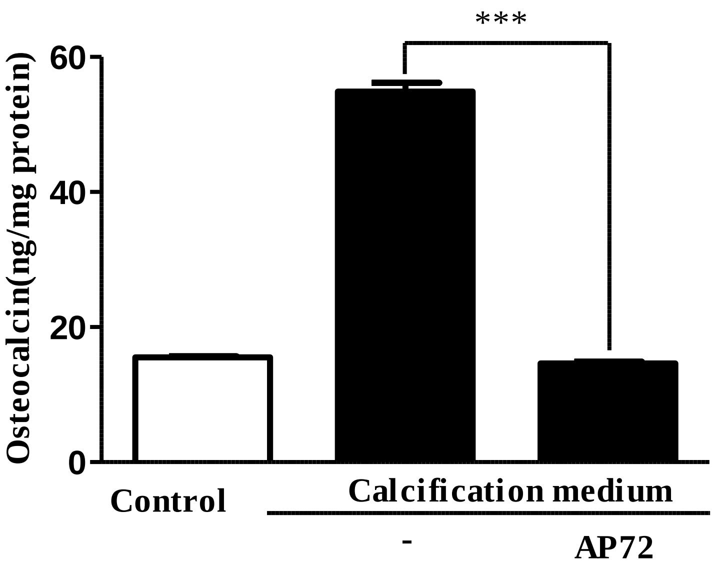

C

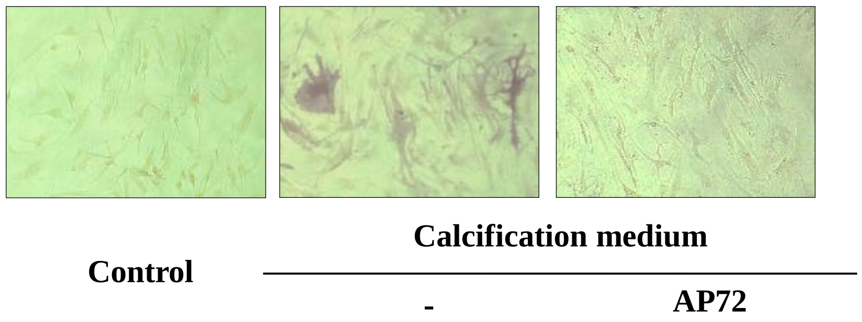

D

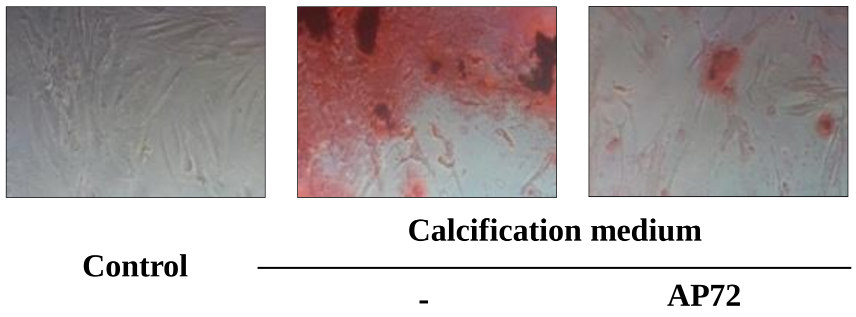

E

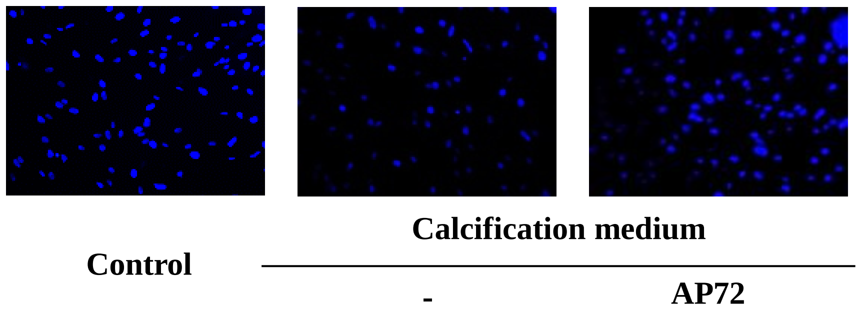

Supplementary Figure 3

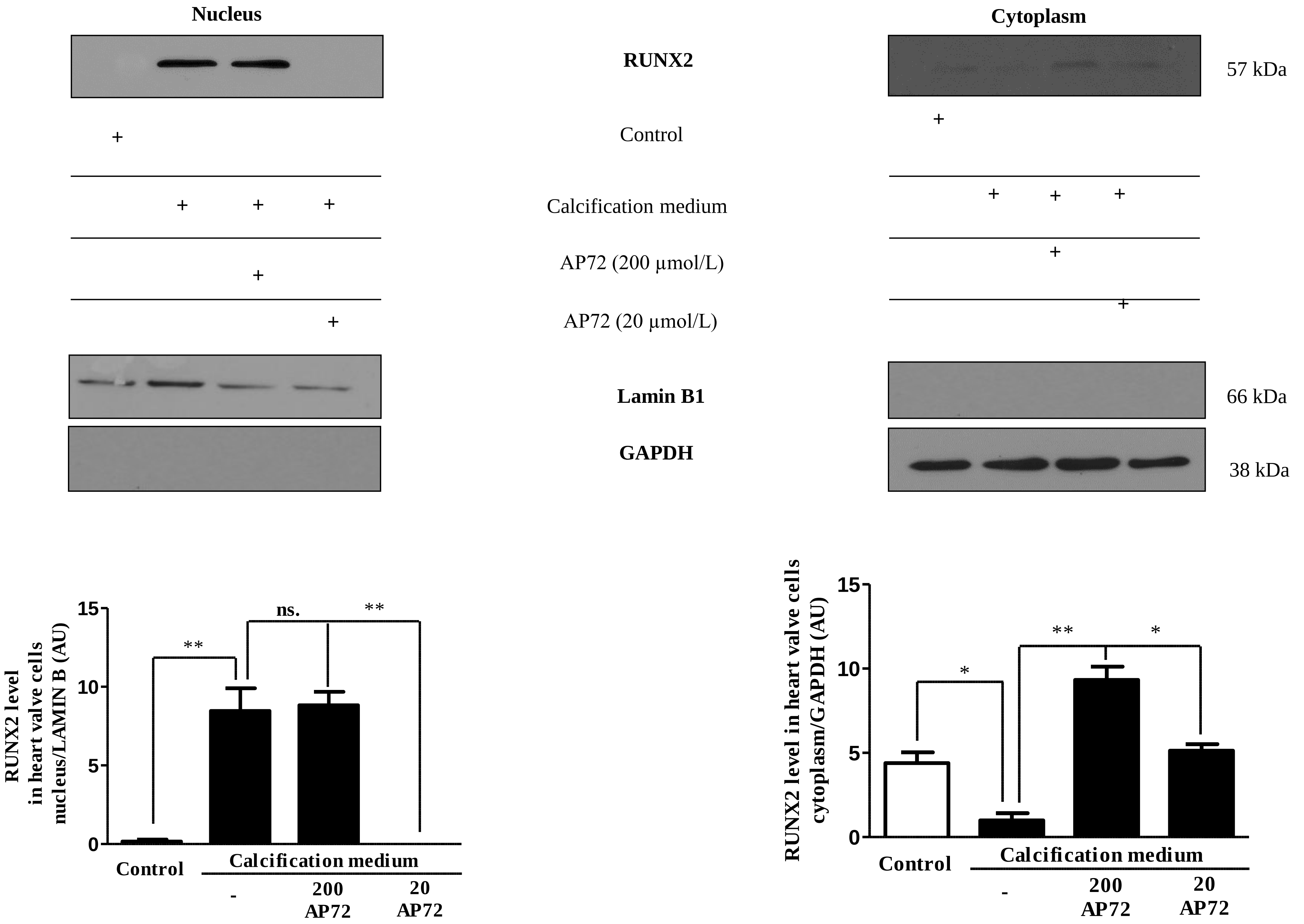

Supplementary Figure 4

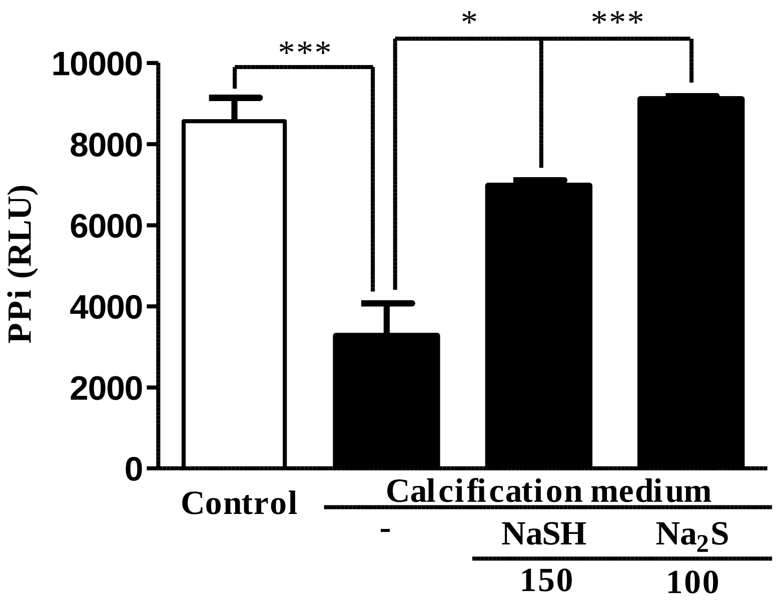

Supplementary Figure 5

A

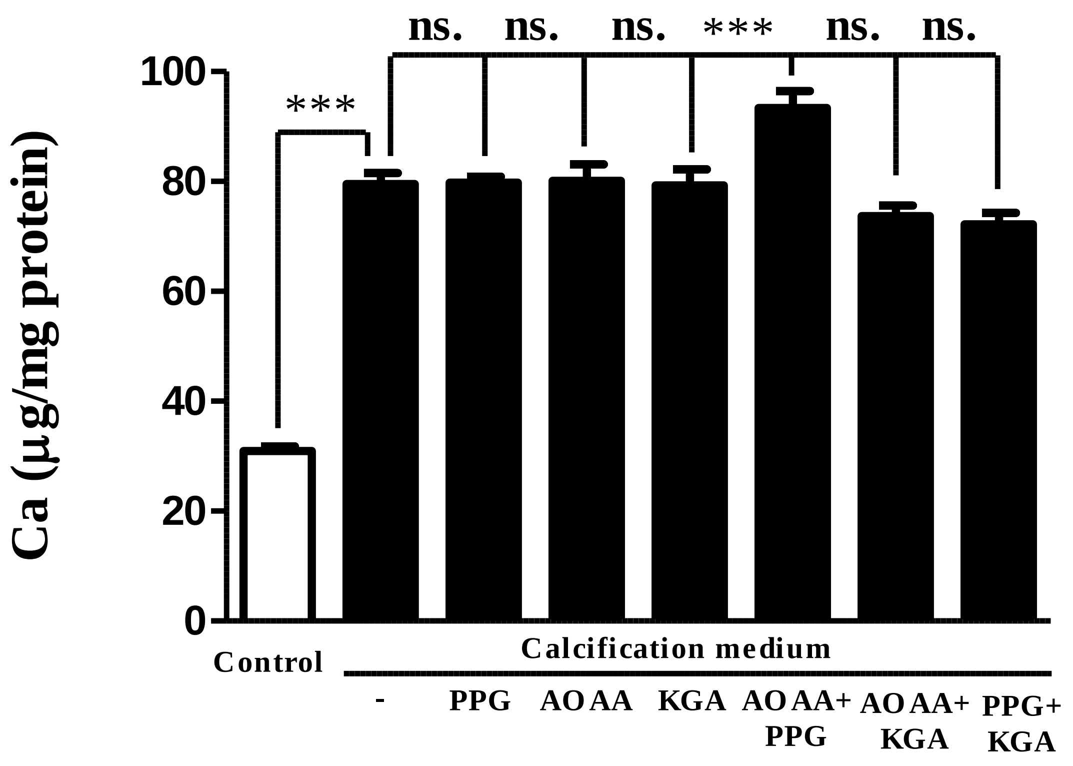

B

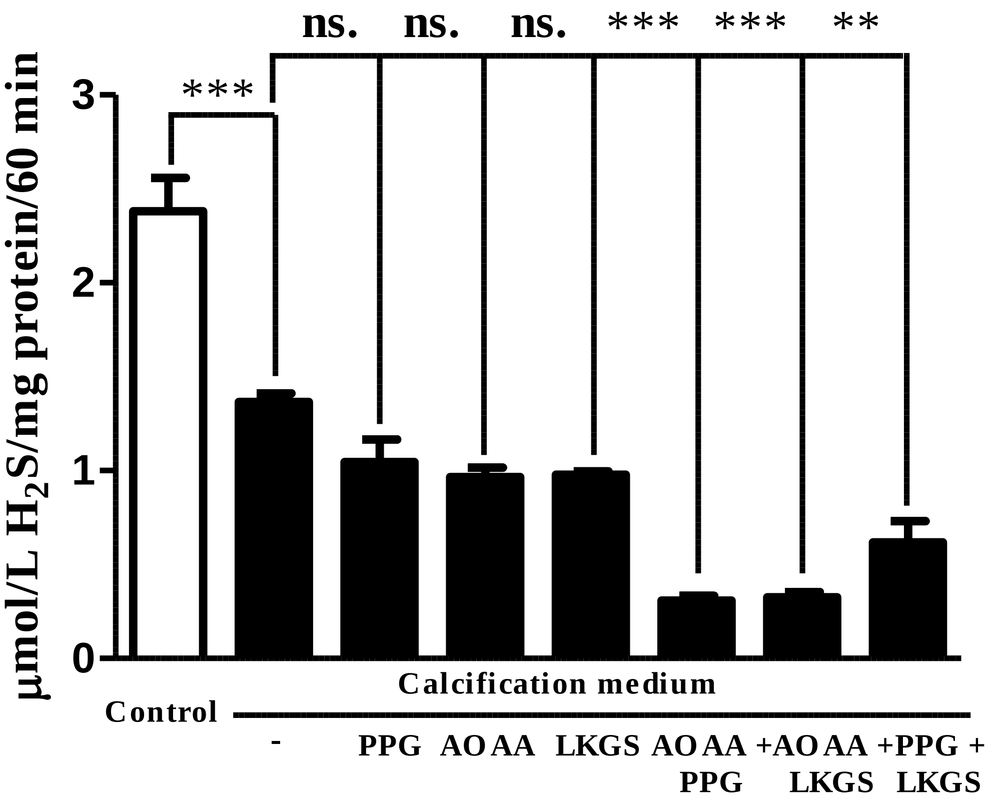

Supplementary Figure 6

A

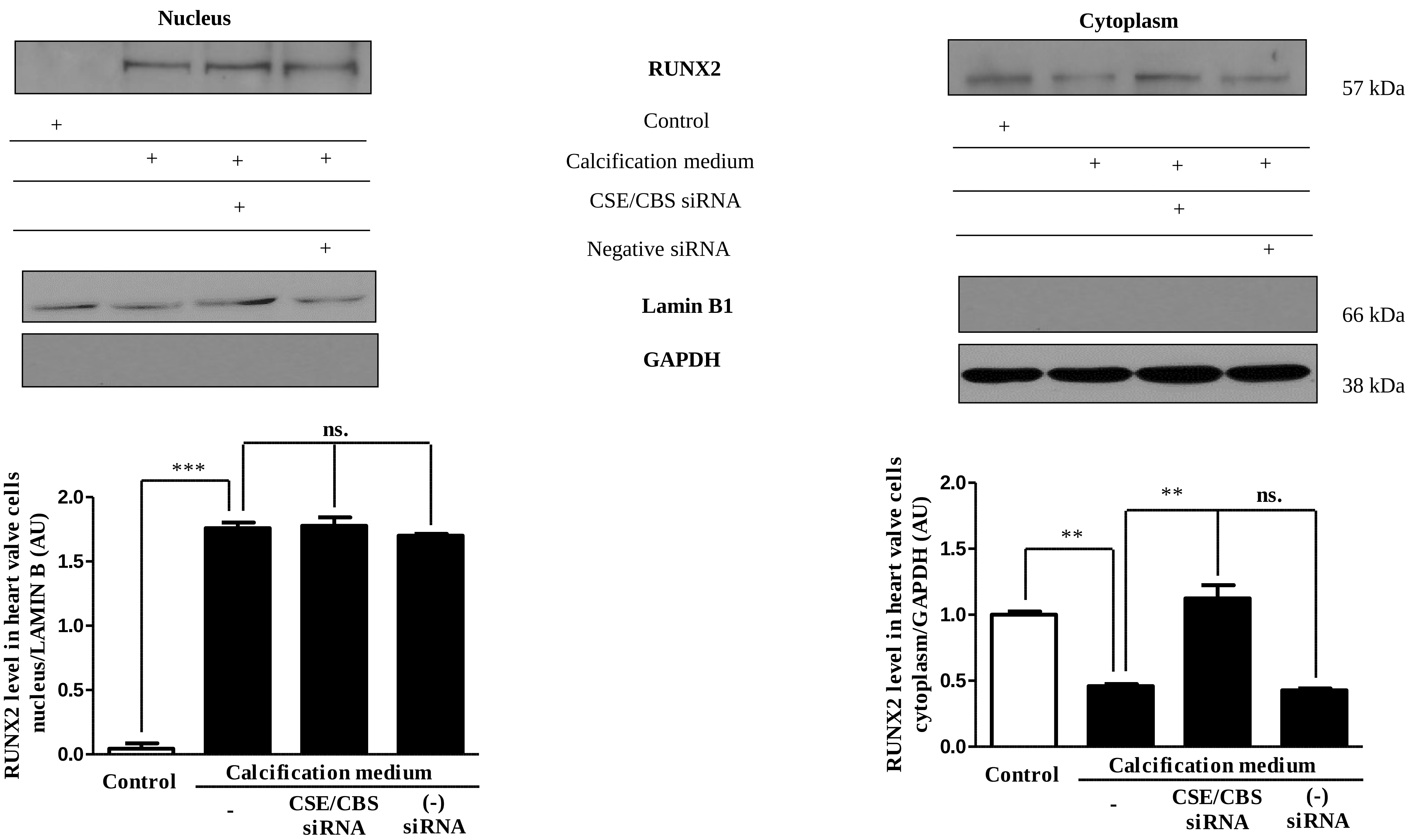

B

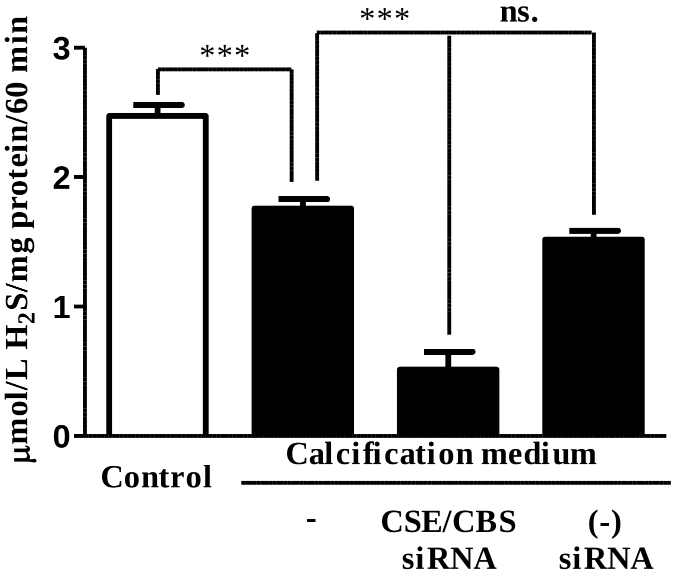

C

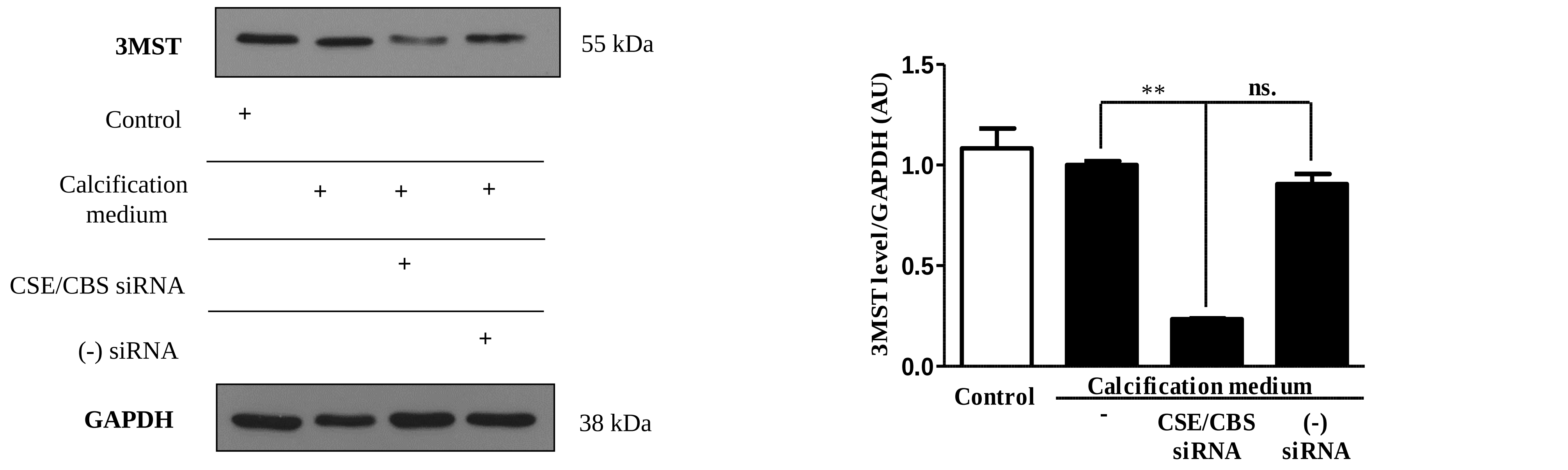

Supplementary Figure 7

A

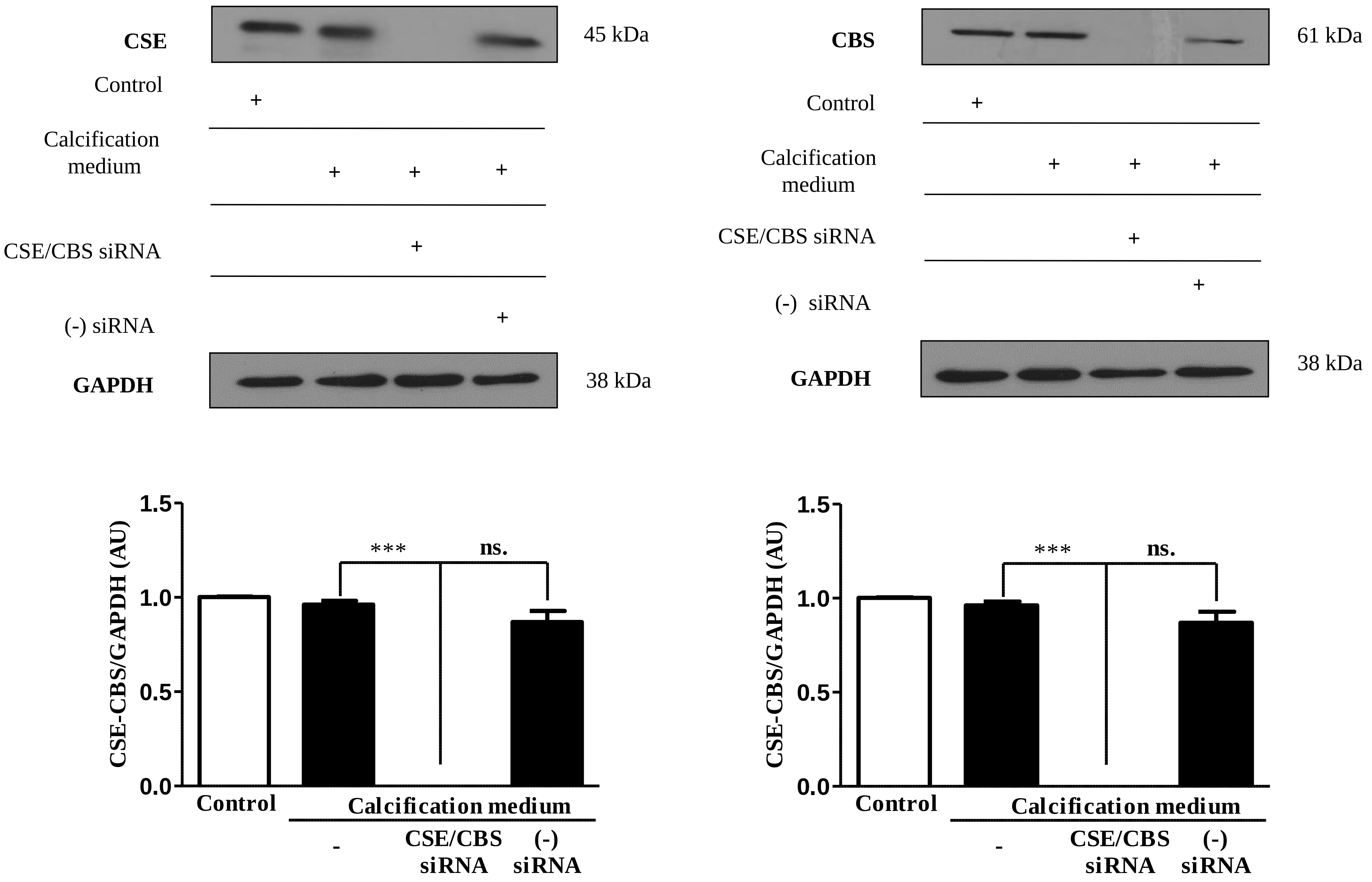

B

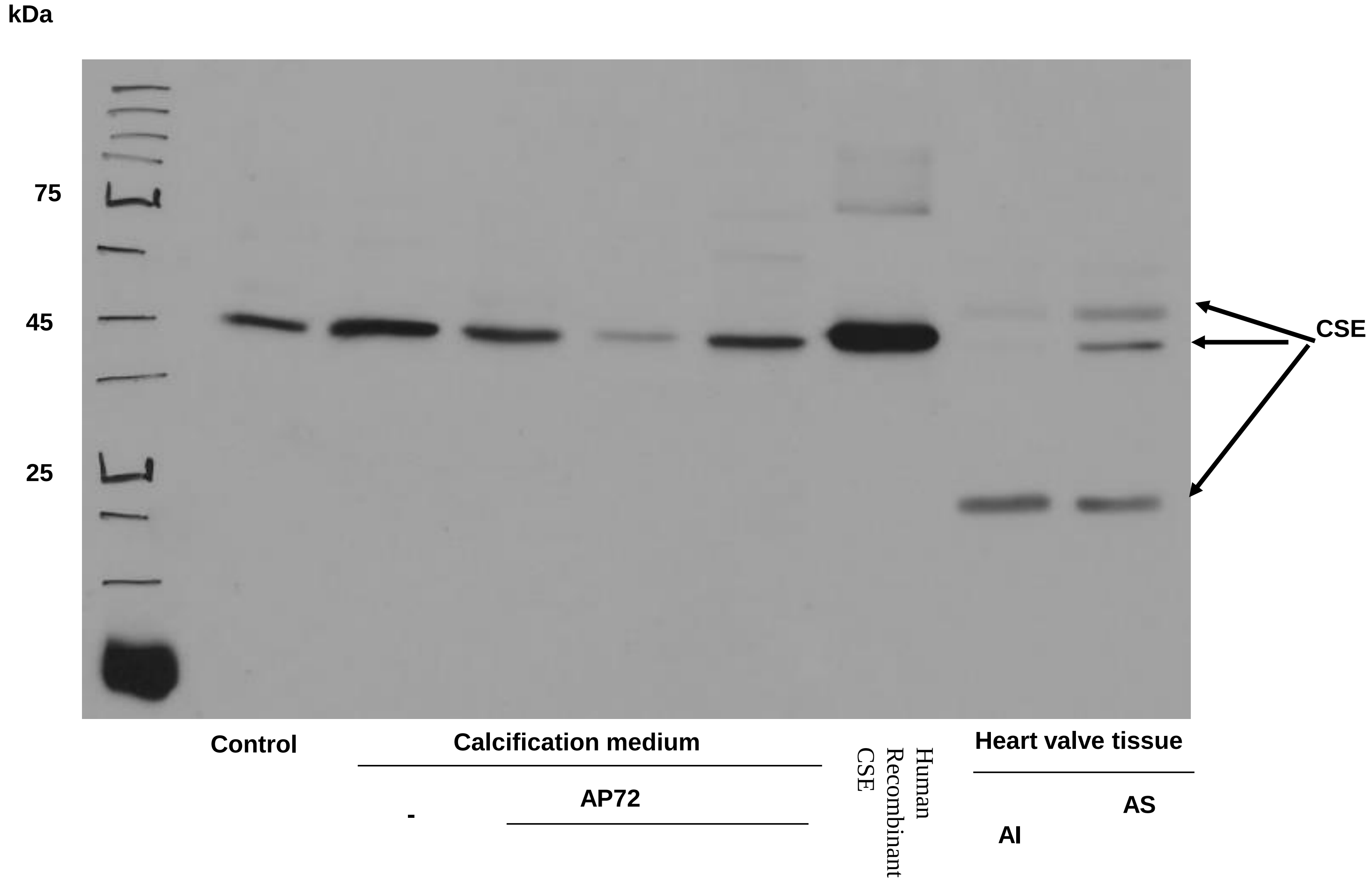

Supplementary Figure 8

SUPPLEMENTARY FIGURE LEGENDS

Supplementary Figure 1. Osteocalcin levels, calcium deposition and cytotoxicity in VIC

Cells were cultured on 24 well plates for 5 days in growth medium or calcification medium and supplemented with NaSH (150µmol/L); Na_2S (100 µmol/L); GYY4137 (50 µmol/L); AP67 (20 µmol/L); AP72 (200 µmol/L). A) Osteocalcin level of the cells were measured after 5 days and normalized to the protein content of the cells. Graph shows mean ±SEM of five separate experiments. B) VIC was cultured in phenol red-free growth or calcifying conditions or supplemented with AP72 (2.5 nmol/L to 5 µmol/L) and calcium content was measured. C) LDH cytotoxicity in VIC exposed to H_2S donors was assessed. All experiments were performed. Results were shown mean ±SEM at least of six independent experiments. Ns.: not significant; *P < 0.05; ***P < 0.001.

Supplementary Figure 2. In phenol red-free condition AP72 inhibits valvular calcification at 2 µmol/L

VIC were cultured in growth medium or calcification medium alone (without phenol red) or supplemented with AP72 (2 µmol/L) for 5 days. A) Calcium content and B) osteocalcin level C) Phosphate uptake of the cells were measured after 5 days and normalized to the protein content of the cells. D) Alkaline phosphatase staining, E) Alizarin Red S staining provides representative microscopic images of extracellular calcium depositions. F) Fluorescence staining assay for cytotoxicity was assessed. Scale bar: 50 µm. Graph shows mean ±SEM of five separate experiments. ***P < 0.001.

Supplementary Figure 3. Inhibition of valvular calcification by AP72

VIC was cultured in growth medium or calcification medium alone or supplemented with AP72 (20 µmol/L) for 5 days. A) Calcium content and B) osteocalcin level of the cells were measured after 5 days and normalized with the protein content of the cells. C) Alkaline phosphatase staining and D) Alizarin Red S staining provide representative microscopic images of extracellular calcium depositions. E) Differences in cellular cytotoxicity is visualized using a fluorescence staining assay (see Methods). Scale bar: 50 µm. Graph shows mean ±SEM of five separate experiments. ***P < 0.001.

Supplementary Figure 4. RUNX2 nuclear translocation in calcifying condition in the presence of 20 and 200 µmol/L of AP72

VIC grown on coverslips were exposed to growth medium or calcification medium alone or supplemented with AP72 (20 and 200 µmol/L) for 24 hours. A) RUNX2 levels of cytoplasm and nucleus were determined by Western blot analyses. The band intensities are normalized to Lamin B1 in case of nuclear extracts and for GAPDH in case of cytoplasm extracts. Representative staining of five independent experiments was shown. Ns.: not significant; **P < 0.01; ***P < 0.001.

Supplementary Figure 5. Changes of PPi levels by fast sulfide donor treatment on VIC

VIC were cultured in growth medium or calcification medium in the presence or absence of NaSH or Na_2S for 5 days. PPi levels of VIC were measured and normalized to protein content of the cells. Graph shows mean ±SEM of five separate experiments. *P < 0.05; ***P < 0.001.

Supplementary Figure 6. Inhibition of H_2S production by pharmacological inhibitors in VIC

A) Calcium accumulation of extracellular matrix of VIC maintained in growth medium or calcification medium after 5 days treatment with PPG; AOAA; KGA; AOAA+PPG; AOAA+KGA; PPG+KGA (20 µmol/L each inhibitor). Calcium levels of VIC were normalized to protein content of the cells. B) H_2S production of VIC treated with pharmacological inhibitors alone or in combination was detected. Generation of H_2S was normalized to the protein content of cells. Graph shows mean ±SEM of five separate experiments. Ns.: not significant; ***P < 0.001.

Supplementary Figure 7. CSE/CBS silencing enhanced the progression of calcification and decreased the expression of 3-MST

CSE and CBS double gene silencing using siRNAs were performed. Cells were transfected with siRNA against CSE/CBS. Next day cells were washed and treated with high inorganic phosphate and calcium for 3 days. A) RUNX2 expression in a nuclear and cytoplasmic fraction were shown. B) H_2S production of CSE/CBS silenced samples was measured. C) Western blot demonstrated expression of 3-MST in CSE/CBS silenced VIC. Graph shows mean ±SEM of five independent experiments. Ns.: not significant; **P < 0.01; ***P < 0.001.

Supplementary Figure 8. CSE and CBS expression of the double silenced VIC

A) VIC were cultured in growth or calcification medium and transfected with siRNA for CSE/CBS. Next day VIC were treated with calcification medium for 3 days. CSE (left panel) and CBS (right panel) protein level were measured. Samples were normalized to GAPDH. B) Western blot of the CSE protein of VIC lysate and AI/AS were shown. Human recombinant CSE protein (5 and 10 ng) was used as a positive control. Ns.: not significant; ***P < 0.001.

Sequences of the siRNA

CTH si RNA sequence:

>gnl|UG|Hs#S6091963 Homo sapiens cystathionase (cystathionine gamma-lyase) (CTH), transcript variant 2, mRNA /cds=p(199,1284) /gb=NM_153742 /gi=299473760 /ug=Hs.19904 /len=2008

TCGTGAGACTGCAGACCCGCTAATAAAATCCACCCCAACAATCGCTGTGTGCCGCTTTAG

TGCGCTCGCCGTCGGCTCTACCTGCGTGCTTTAGCTCCTTCTCGCCTGATCCTTCTGTCT

CTCCCAACCCCGGACACCCGGCTTCGACTGGTTATATCTTCGGTGTTCTTTTCCTCTCTT

CTTCTTTCGCGGTTCAGCATGCAGGAAAAAGACGCCTCCTCACAAGGTTTCCTGCCACAC

TTCCAACATTTCGCCACGCAGGCGATCCATGTGGGCCAGGATCCAGAGCAATGGACCTCC

AGGGCTGTAGTGCCCCCCATCTCACTGTCCACCACGTTCAAGCAAGGGGCGCCTGGCCAG

CACTCGGGTTTTGAATATAGCCGTTCTGGAAATCCCACTAGGAATTGCCTTGAAAAAGCA

GTGGCAGCACTGGATGGGGCTAAGTACTGTTTGGCCTTTGCTTCAGGTTTAGCAGCCACT

GTAACTATTACCCATCTTTTAAAAGCAGGAGACCAAATTATTTGTATGGATGATGTGTAT

GGAGGTACAAACAGGTACTTCAGGCAAGTGGCATCTGAATTTGGATTAAAGATTTCTTTT

GTTGATTGTTCCAAAATCAAATTACTAGAGGCAGCAATTACACCAGAAACCAAGCGCCCT

TTGGCTCTGGGAGCTGATATTTCTATGTATTCTGCAACAAAATACATGAATGGCCACAGT

GATGTTGTAATGGGCCTGGTGTCTGTTAATTGTGAAAGCCTTCATAATAGACTTCGTTTC

TTGCAAAACTCTCTTGGAGCAGTTCCATCTCCTATTGATTGTTACCTCTGCAATCGAGGT

CTGAAGACTCTACATGTCCGAATGGAAAAGCATTTCAAAAACGGAATGGCAGTTGCCCAG

TTCCTGGAATCTAATCCTTGGGTAGAAAAGGTTATTTATCCTGGGCTGCCCTCTCATCCA

CAGCATGAGTTGGTGAAGCGTCAGTGTACAGGTTGTACAGGGATGGTCACCTTTTATATT

AAGGGCACTCTTCAGCATGCTGAGATTTTCCTCAAGAACCTAAAGCTATTTACTCTGGCC

GAGAGCTTGGGAGGATTCGAAAGCCTTGCTGAGCTTCCGGCAATCATGACTCATGCATCA

GTTCTTAAGAATGACAGAGATGTCCTTGGAATTAGTGACACACTGATTCGACTTTCTGTG

GGCTTAGAGGATGAGGAAGACCTACTGGAAGATCTAGATCAAGCTTTGAAGGCAGCACAC

CCTCCAAGTGGAAGTCACAGCTAGTATTCCAGAGCTGCTATTAGAAGCTGCTTCCTGTGA

AGATCAAATCTTCCTGAGTAATTAAATGGACCAACAATGAGCCTTTGCAAAATTTTCAAG

CGGAAATTTTAAGGCACCTCATTATCTTTCATAACTGTAATTTTCTTAGGGATCATCTCT

GTTAAAAAGTTTTCTGTATGTCATGTTATAATTACAGGTCAATTCTGTTAATATCTTTTT

GTTAATTTTGCTCTATGTTTGCCTCTGAAGGAGGTGAGATTTGTGCTACTTTGGGAGATT

ATGTTCTTTTTTCATGTCTAAGATTTATTTTGATCATGTTTATAATATAATGGTAATTCA

TTTTTGATGTTTTGTGAAGAATTTAAATTTAAACGAATGTTCTTAAATCAAGTGTGATTT

TTTTGCATATCATTGAAAAGAACATTAAAAGCAATGGTTTACACTTAGTTACCATAAGCC

GAAAATCAAATACTTGAAAAGTTTACTGTGAAATTCTACTGATTTAAGACTATACTTAAT

ATTTTTAAAAAAATAAATCAGCTGGGCGCGGTGGCTCACGCATGTAATGCCAGCACTTTT

GGAGGATAAGGCGGGCGGATCACGAGGTCAGGAGATTGAGACCATCCTGGCTAGCGCAGT

GAAACCCCCATCTCTACTAAAAATGCAAAAAAAATTAGACGGACGTGGTGGCGGGTGCCT

GTAGTCCCAGCTACTTGGGAGGCTGAGG

CBS siRNA sequence

>gnl|UG|Hs#S55481398 Homo sapiens cystathionine-beta-synthase (CBS), transcript variant 2, mRNA /cds=p(223,1878) /gb=NM_001178008 /gi=295821199 /ug=Hs.533013 /len=2586

CACGCCCTCGGGGTCGGTCCTCGAGGACGCGCAGGGCCCCCACCCACCAGGACGCACGT

TTCAAGCTCATCAGTAAAGGTTCCTTAAATTCCCGAAGGGCAAGAAGTTAACCAAGTAAA

ACAGCATCGGAACACCAGGATCCCATGACAGATTCTGTTGTCACGTCTCCTTACAGAGTT

TGAGCGGTGCTGAACTGTCAGCACCATCTGTCCGGTCCCAGCATGCCTTCTGAGACCCCC

CAGGCAGAAGTGGGGCCCACAGGCTGCCCCCACCGCTCAGGGCCACACTCGGCGAAGGGG

AGCCTGGAGAAGGGGTCCCCAGAGGATAAGGAAGCCAAGGAGCCCCTGTGGATCCGGCCC

GATGCTCCGAGCAGGTGCACCTGGCAGCTGGGCCGGCCTGCCTCCGAGTCCCCACATCAC

CACACTGCCCCGGCAAAATCTCCAAAAATCTTGCCAGATATTCTGAAGAAAATCGGGGAC

ACCCCTATGGTCAGAATCAACAAGATTGGGAAGAAGTTCGGCCTGAAGTGTGAGCTCTTG

GCCAAGTGTGAGTTCTTCAACGCGGGCGGGAGCGTGAAGGACCGCATCAGCCTGCGGATG

ATTGAGGATGCTGAGCGCGACGGGACGCTGAAGCCCGGGGACACGATTATCGAGCCGACA

TCCGGGAACACCGGGATCGGGCTGGCCCTGGCTGCGGCAGTGAGGGGCTATCGCTGCATC
ATCGTGATGCCAGAGAAGATGAGCTCCGAGAAGGTGGACGTGCTGCGGGCACTGGGGGCT
GAGATTGTGAGGACGCCCACCAATGCCAGGTTCGACTCCCCGGAGTCACACGTGGGGGTG
GCCTGGCGGCTGAAGAACGAAATCCCCAATTCTCACATCCTAGACCAGTACCGCAACGCC
AGCAACCCCCTGGCTCACTACGACACCACCGCTGATGAGATCCTGCAGCAGTGTGATGGG
AAGCTGGACATGCTGGTGGCTTCAGTGGGCACGGGCGGCACCATCACGGGCATTGCCAGG
AAGCTGAAGGAGAAGTGTCCTGGATGCAGGATCATTGGGGTGGATCCCGAAGGGTCCATC
CTCGCAGAGCCGGAGGAGCTGAACCAGACGGAGCAGACAACCTACGAGGTGGAAGGGATC
GGCTACGACTTCATCCCCACGGTGCTGGACAGGACGGTGGTGGACAAGTGGTTCAAGAGC
AACGATGAGGAGGCGTTCACCTTTGCCCGCATGCTGATCGCGCAAGAGGGGCTGCTGTGC
GGTGGCAGTGCTGGCAGCACGGTGGCGGTGGCCGTGAAGGCCGCGCAGGAGCTGCAGGAG
GGCCAGCGCTGCGTGGTCATTCTGCCCGACTCAGTGCGGAACTACATGACCAAGTTCCTG
AGCGACAGGTGGATGCTGCAGAAGGGCTTTCTGAAGGAGGAGGACCTCACGGAGAAGAAG
CCCTGGTGGTGGCACCTCCGTGTTCAGGAGCTGGGCCTGTCAGCCCCGCTGACCGTGCTC
CCGACCATCACCTGTGGGCACACCATCGAGATCCTCCGGGAGAAGGGCTTCGACCAGGCG
CCCGTGGTGGATGAGGCGGGGGTAATCCTGGGAATGGTGACGCTTGGGAACATGCTCTCG
TCCCTGCTTGCCGGGAAGGTGCAGCCGTCAGACCAAGTTGGCAAAGTCATCTACAAGCAG
TTCAAACAGATCCGCCTCACGGACACGCTGGGCAGGCTCTCGCACATCCTGGAGATGGAC
CACTTCGCCCTGGTGGTGCACGAGCAGATCCAGTACCACAGCACCGGGAAGTCCAGTCAG
CGGCAGATGGTGTTCGGGGTGGTCACCGCCATTGACTTGCTGAACTTCGTGGCCGCCCAG
GAGCGGGACCAGAAGTGAAGTCCGGAGCGCTGGGCGGTGCGGAGCGGGCCCGCCACCCTT
GCCCACTTCTCCTTCGCTTTCCTGAGCCCTAAACACACGCGTGATTGGTAACTGCCTGGC
CTGGCACCGTTATCCCTGCACACGGCACAGAGCATCCGTCTCCCCTCGTTAACACATGGC
TTCCTAAATGGCCCTGTTTACGGCCTATGAGATGAAATATGTGATTTTCTCTAATGTAAC
TTCCTCTTAGGATGTTTCACCAAGGAAATATTGAGAGAGAAGTCGGCCAGGTAGGATGAA
CACAGGCAATGACTGCGCAGAGTGGATTAAAGGCAAAAGAGAGAAGAGTCCAGGAAGGGG

CGGGGAGAAGCCTGGGTGGCTCAGCATCCTCCACGGGCTGCGCCGTCTGCTCGGGGCTGA

GCTGGCGGGAGCAGTTTGCGTGTTTGGGTTTTTTAATTGAGATGAAATTCAAATAACCTA

AAAATCAATCACTTGAAAGTGAACAATCAGCGGCATTTAGTACATCCAGAAAGTTGTGTA

GGCACCACCTCTGTCACGTTCTGGAACATTCTGTCATCACCCCGTGAAGCAATCATTTCC

CCTCCCGTCTTCCTCCTCCCCTGGCAACTGCTGATCGACTTTGTGTCTCTGTTGTCTAAA

ATAGGTTTTCCCTGTTCTGGACATTTCATATAAATGGAATCACACAAAAAAAAAAAAAAA

AAAAAA.
